# TDGF1 Mediates the Oncogenic Effects of the OLMALINC/miR-3614-5p ceRNA Axis in Colon Cancer Through Nodal/Smad2 and Glypican-1/MAPK-AKT Signaling

**DOI:** 10.3390/cells15131141

**Published:** 2026-06-23

**Authors:** Feng Gao, Xiaoli Li, Jiawei Li, Shuo Yang, Boyu Zhang, Ying Sun, Lihua Zheng, Guannan Wang, Lei Liu, Yongli Bao, Xiaoguang Yang

**Affiliations:** 1 National Engineering Laboratory for Druggable Gene and Protein Screening, Northeast Normal University, Changchun 130117, China; 2 International Joint Research Center of Stem Cell Bank, Ministry of Science and Technology, Northeast Normal University, Changchun 130117, China

**Keywords:** colon cancer, TDGF1, ncRNAs, signaling pathway, prognosis, immune signature

## Abstract

**Highlights:**

**What are the main findings?**

- OLMALINC sponges miR-3614-5p to upregulate TDGF1, driving colon cancer progression via Nodal/Smad2 and MAPK/AKT pathways.
- High TDGF1 expression correlates with an immunosuppressive microenvironment, advanced tumor stage, and poor prognosis.

**What is the implication of the main finding?**

- TDGF1 serves as a promising prognostic biomarker for predicting clinical outcomes in colon cancer patients.
- The OLMALINC/miR-3614-5p/TDGF1 regulatory axis provides potential targets for future therapeutic strategies.

**Abstract:**

The multifaceted oncogenic role of teratocarcinoma-derived growth factor 1 (TDGF1) in colon cancer remains incompletely understood. Through integrative bioinformatic and functional analyses, we identified a novel competing endogenous RNA (ceRNA) axis wherein the long non-coding RNA OLMALINC directly sponges hsa-miR-3614-5p, leading to the derepression of TDGF1. This OLMALINC/miR-3614-5p/TDGF1 axis promoted colon cancer cell proliferation, migration, invasion, and anti-apoptosis in vitro, whereas TDGF1 knockdown significantly suppressed tumor growth in vivo. Mechanistically, TDGF1 co-activated oncogenic signaling via the Thr88-dependent Nodal/Smad2 cascade and the Glypican-1-mediated MAPK/AKT pathway. Beyond cell-autonomous effects, transcriptomic and single-cell analyses revealed that elevated TDGF1 correlates with an immunosuppressive microenvironment, characterized by reduced immune infiltration and altered LGALS9-CD44 malignant-T cell communication. Clinically, high TDGF1 expression in a tissue microarray cohort was significantly associated with advanced T stage, reduced expression of specific mismatch repair proteins (MLH1/PMS2), and poor overall survival. Collectively, this study delineates the OLMALINC/miR-3614-5p/TDGF1 regulatory circuit and establishes TDGF1 as a multifaceted driver of tumor progression, highlighting its potential as a prognostic biomarker and therapeutic target in colon cancer.

## 1. Introduction

Globally, colon cancer represents the fifth most prevalent malignancy and the fifth leading cause of cancer-related mortality [1]. Colon adenocarcinoma (COAD) constitutes the most frequent pathological subtype [2]. Both the incidence and mortality rates of colon cancer demonstrate a persistent annual increase [1]. Current therapeutic strategies rely on comprehensive treatment involving surgery combined with adjuvant chemotherapy, which has significantly improved patient 5-year survival rates. However, a critical challenge persists as the majority of colon cancer patients present with late-stage diagnoses, frequently experiencing post-treatment recurrence and metastasis [3,4]. Consequently, identifying effective therapeutic targets or discovering robust prognostic biomarkers for colon cancer remains an urgent unmet need.

Notably, Teratocarcinoma-derived growth factor 1 (TDGF1, also termed as Cripto-1), a member of the EGF/CFC family, plays a vital role in embryogenesis [5,6]. Extensive evidence confirms that TDGF1 is overexpressed in numerous human carcinomas, correlating significantly with enhanced cell proliferation, migration, invasion, and epithelial to mesenchymal transition (EMT), including breast cancer [7,8,9], lung cancer [10,11], gastrointestinal tract cancers [12,13,14,15,16], liver cancer [17,18], renal cancer [19], reproductive system cancers [20,21,22,23,24], and cutaneous melanoma [25]. In the serum of patients with colon cancer, a significantly higher TDGF1 level is detected compared to healthy individuals [16]. Consistently, immunohistochemical analyses reveal abundant TDGF1 expression in colon cancer tissues, where its levels positively correlate with tumor size, lymph node metastasis, depth of invasion, liver metastasis, and advanced TNM stage. Furthermore, patients exhibiting high TDGF1 expression demonstrate significantly poorer overall survival (OS) and reduced disease-free survival (DFS) [13]. Mechanistically, TDGF1 knockdown suppresses colorectal cancer cell proliferation and migration via inhibition of the Akt and MAPK signaling pathways [13]. Nevertheless, a systematic analysis encompassing TDGF1 expression dynamics, prognostic value, mechanistic underpinnings, and crucially, its role within the colon cancer immune microenvironment, is still lacking.

To address these gaps, we comprehensively analyzed TDGF1 expression patterns across diverse human cancers and evaluated its association with tumorigenesis and patient survival outcomes. Subsequently, the potential regulatory noncoding RNAs (ncRNAs), including microRNAs (miRNAs) and long noncoding RNAs (lncRNAs), involved in the regulation of TDGF1, were explored in colon cancer. Next, we investigated correlations between TDGF1 expression and immune cell infiltration, immune cell biomarkers, and immune checkpoint molecules within the colon cancer tumor immune microenvironment (TIME). Finally, leveraging bulk RNA sequencing and single-cell RNA sequencing (scRNA-seq) data, we delineated TDGF1-associated biological pathways, constructed cell–cell interaction networks, and identified interacting proteins within these interacting cells. Meanwhile, the association between TDGF1 expression and clinicopathological characteristics of colon cancer was analyzed. Collectively, our findings provide additional references elucidating TDGF1’s multifaceted role in colon cancer pathogenesis and immunity.

## 2. Materials and Methods

### 2.1. TCGA Database Analysis

Transcriptomic data for COAD were retrieved from The Cancer Genome Atlas (TCGA) project via the GDC Data Portal (https://portal.gdc.cancer.gov/, accessed on 16 April 2022). Bioinformatics analyses were conducted using the Xiantao Academic Platform (https://www.xiantaozi.com/, accessed on 28 June 2025). Level 3 RNA-sequencing data were normalized to transcripts per million (TPM) and subsequently log_2_-transformed for all downstream comparisons. Differential expression of miRNAs and lncRNAs between tumor and adjacent normal tissues was assessed. The diagnostic potential of TDGF1 and candidate non-coding RNAs was evaluated using receiver operating characteristic (ROC) curve analysis, with the area under the curve (AUC) ranging from 0.5 (no discrimination) to 1.0 (perfect discrimination). Forest plots were generated to visualize the prognostic associations of TDGF1, hsa-miR-3614-5p, and OLMALINC. As this study exclusively utilized publicly available, de-identified data from TCGA, it did not constitute human subjects research under the project’s Data Use Policy; therefore, no additional ethical approval or patient consent was required.

### 2.2. GEPIA Database Analysis

The GEPIA database (http://gepia2.cancer-pku.cn/#analysis, accessed on 31 March 2022) [26] was employed to: (i) compare TDGF1 expression between tumor and paired normal tissues, (ii) conduct survival analyses (OS/DFS) across cancer types, and (iii) evaluate TDGF1 association with pathological stages in COAD and READ. Furthermore, GEPIA facilitated correlation analysis between TDGF1 and immune cell biomarkers/checkpoints in colon cancer.

### 2.3. TISIDB Database Analysis

Tumor-immune system interactions were interrogated via the TISIDB database [27], which integrates multiple heterogeneous data types. Specifically, we used the TISIDB database to analyze the expression of TDGF1 in COAD and READ subtypes.

### 2.4. miRNA Prediction Database Analysis

Regarding miRNA regulation, putative TDGF1-targeted miRNAs were predicted using TargetScan (https://www.targetscan.org/vert_80/, accessed on 26 March 2022) [28], miRDB (http://mirdb.org/, accessed on 27 March 2022) [29], miRmap (https://mirmap.ezlab.org/, accessed on 27 March 2022) [30], miRNA Target (www.mirnatarget.com/, accessed on 27 March 2022), and miRWalk (http://mirwalk.umm.uni-heidelberg.de/, accessed on 30 March 2022/) [31] computational platforms. To ensure robustness, miRNAs identified in more than three platforms were retained for validation.

### 2.5. ENCORI Database Analysis

For lncRNA-miRNA interactions, the ENCORI database (https://starbase.sysu.edu.cn/, accessed on 5 April 2022) [32] was utilized to predict candidate lncRNAs that could potentially bind to target miRNAs. Concurrently, ENCORI was further introduced to perform expression correlations between miRNAs and TDGF1 in colon cancer.

### 2.6. TIMER Database Analysis

TIMER (https://cistrome.shinyapps.io/timer/, accessed on 13 April 2022) [33], as an online server, is often used to comprehensively analyze immune infiltration in multiple types of tumors. In this study, we utilized the TIMER database to determine the correlations between TDGF1 expression and immune cell/subtype infiltration levels or immune checkpoint expression in colon cancer.

### 2.7. LinkedOmics Database

For co-expression network analysis, the LinkedOmics (http://www.linkedomics.org/login.php, accessed on 14 April 2022) [34] database -housing 32 TCGA cancer types and 10 clinical proteomics tumor analysis consortium (CPTAC) cancer cohorts, identified TDGF1-co-expressed genes. Subsequently, the Gene Ontology biological process (GO_BP) and KEGG pathways of TDGF1 and its co-expression genes were explored using gene set enrichment analysis (GSEA).

### 2.8. STRING Database

The STRING database (https://string-db.org/, accessed on 14 April 2022) [35] contains publicly available sources of protein–protein interactions, which were queried for TDGF1-binding proteins.

### 2.9. CancerSCEM Database Analysis

CancerSCEM database (https://ngdc.cncb.ac.cn/cancerscem/index, accessed on 19 April 2022) [36] is a public database for visualizing single-cell RNA-sequencing data of human cancers. The database was leveraged to: (i) visualize TDGF1 distribution/expression across single-cell populations, (ii) identify TDGF1 co-expressed genes in malignant cells, (iii) construct cell–cell interaction networks, and (iv) verify interacting proteins within the interacting cells. Notably, TDGF1 co-expressed genes in more than two single-cell samples were selected for display.

### 2.10. Metascape Database Analysis

Metascape (https://metascape.org/gp/index.html#/main/step1, accessed on 20 April 2022) [37], a web-based portal for comprehensive gene annotation and analysis, was employed for functional annotation of TDGF1-associated differentially expressed genes (DEGs) in malignant cells. Additionally, protein–protein interaction (PPI) networks were investigated using the platform.

### 2.11. Cell Culture and Transfection

HT-29 and HCT-116 human colon cancer cells were obtained from the Shanghai Institute of Biosciences Cell Resource Center, Chinese Academy of Sciences (Shanghai, China). Cells were maintained in RPMI 1640 (Corning, NY, USA) supplemented with serum (10%), penicillin (100 U/mL), and streptomycin (0.1 mg/mL) at 37 °C in a humidified 5% CO_2_ incubator. The siRNAs targeting OLMALINC (5′-GAGTCAGCAAAACACACTA-3′), TDGF1 (5′-CAGCACAGTAAGGAGCTAA-3′), and a non-targeting control were synthesized by Genepharma (Suzhou, China). A short hairpin RNA (shRNA) targeting Glypican-1 (shGPC1, 5′-GCTGGTCTACTGTGCTCAC-3′) was constructed by the MiaoLing Plasmid Platform (Wuhan, China). For overexpression, the coding sequences of OLMALINC, flag-TDGF1, flag-TDGF1-T88A mutant, myc-Nodal, and his-Glypican-1 were cloned into expression vectors by the same platform. Transient transfection of siRNAs and plasmids was performed using Lipofectamine 2000 (Invitrogen, Shanghai, China) according to the manufacturer’s protocol.

### 2.12. Luciferase Reporter Assay

The potential binding sites of hsa-miR-3614-5p with OLMALINC or TDGF1 were predicted using the ENCORI and TargetScan online tools. Subsequently, reporter vectors were generated by cloning sequences containing either the wild-type (wt) or mutant (mut) binding sites into the pmirGLO vector (Promega, USA). These constructs were then co-transfected into HEK 293T cells alongside hsa-miR-3614-5p mimics or a control mimic (RiboBio, Guangzhou, China). After 48 h, luciferase activity was quantified using the Dual-Luciferase Reporter Assay System (Promega, Madison, WI, USA).

### 2.13. Nuclear/Cytoplasmic Fractionation

Approximately 10^7^ cells were harvested, resuspended in 1 mL of ice-cold RNase-free PBS, 1 mL of buffer C1 (1.28 M sucrose, 40 mM Tris-HCl [pH 7.5], 20 mM MgCl_2_, 4% Triton X-100) and 3 mL of RNase-free water, then incubated on ice for 15 min. Cells were subsequently centrifuged at 2500 rpm for 15 min. The supernatant, containing the cytoplasmic fraction, and the nuclear pellet were both retained for RNA extraction.

### 2.14. Quantitative Real-Time PCR

Total RNA was extracted from transfected cells using TRIzol reagent (Invitrogen, Carlsbad, CA, USA). Subsequently, quantitative real-time PCR (qRT-PCR) was performed with a SYBR Green kit (TransGen, Beijing, China) following the manufacturer’s protocol. All qRT-PCR primer sequences are listed in Table S1. Relative gene expression levels were calculated using the 2^−ΔΔCt^ method, with GAPDH or U6 expression serving as internal controls for normalization.

### 2.15. Western Blot

Total protein was extracted from tumor cells with RIPA lysis buffer, and protein concentrations were quantified using a BCA assay kit (Beyotime, Shanghai, China). Proteins were resolved by 10% SDS-PAGE and electrophoretically transferred onto PVDF membranes. Membranes were then blocked with 5% skim milk and incubated overnight at 4 °C with primary antibodies against TDGF1 (HY-P991640, MCE, Shanghai, China), flag (66008-4-Ig, Proteintech, Wuhan, China), his (66005-1-Ig, Proteintech, Wuhan, China), myc (60003-2-Ig, Proteintech, Wuhan, China), H3 (17168-1-AP, Proteintech, Wuhan, China), AKT (10176-2-AP, Proteintech, Wuhan, China), p-AKT (80455-1-RR, Proteintech, Wuhan, China), MAPK (11257-1-AP, Proteintech, Wuhan, China), p-MAPK (28733-1-AP, Proteintech, Wuhan, China), smad2 (12570-1-AP, Proteintech, Wuhan, China), p-smad2 (80427-2-RR, Proteintech, Wuhan, China), Glypican-1 (R22799, Zenbio, Chengdu, China), E-cadherin (20874-1-AP, Proteintech, Wuhan, China), N-cadherin (22018-1-AP, Proteintech, Wuhan, China), Vimentin (10366-1-AP, Proteintech, Wuhan, China), Snail (sc-271977, Santa Cruz, TX, USA), Slug (12129-1-AP, Proteintech, Wuhan, China), BAX (50599-2-Ig, Proteintech, Wuhan, China), BCL2 (12789-1-AP, Proteintech, Wuhan, China), Cleaved Caspase-3 (25128-1-AP, Proteintech, Wuhan, China), Cleaved Caspase-9 (9509T, CST, Boston, MA, USA), MLH1(11697-1-AP, Proteintech, Wuhan, China), PMS2(66075-1-Ig, Proteintech, Wuhan, China), and GAPDH (10494-1-AP, Proteintech, Wuhan, China). After washing three times with TBST, membranes were incubated with horseradish peroxidase (HRP)-conjugated secondary antibodies. Protein bands were visualized using an enhanced chemiluminescence (ECL) detection system (Boster, Wuhan, China), with GAPDH serving as the loading control for normalization.

### 2.16. Co-Immunoprecipitation Assay

Cells cultured to 80-90% confluence were lysed on ice using immunoprecipitation buffer supplemented with protease inhibitor (both from Beyotime, Shanghai, China). After centrifugation, the supernatant was collected and incubated with either an anti-flag primary antibody (1:100) or normal IgG (1:100, Beyotime, Shanghai, China) at 4 °C overnight. Subsequently, protein A/G magnetic beads (MCE, Shanghai, China) were added and incubated for 4 h. The beads were then thoroughly washed, and the bound proteins were eluted by boiling in SDS-PAGE loading buffer for subsequent Western blot analysis.

### 2.17. MTT Assay

Cell viability was dynamically monitored via MTT assay at 72 h. Cells were plated in 96-well plates under standard culture conditions, with absorbance quantified according to the manufacturer’s specifications.

### 2.18. Colony Formation Assay

Transfected cells were seeded into 6-well plates and cultured at 37 °C with 5% CO_2_ for 14 days. The culture medium was replaced with fresh medium every 72 h. Colonies were fixed and stained with 0.1% crystal violet, followed by solubilization in 33% glacial acetic acid. Absorbance of the solubilized dye was measured at 570 nm using a spectrophotometer.

### 2.19. Transwell Assay

Cell migration and invasion capacities were assessed using transwell chambers. For migration assays, 1 × 10^4^ cells in serum-free medium were seeded into the upper chamber, while the lower chamber contained complete medium supplemented with 10% FBS. After 24 h incubation at 37 °C, migrated cells were fixed with 4% paraformaldehyde and stained with 0.1% crystal violet. Following solubilization in 33% glacial acetic acid, absorbance was measured at 570 nm using a spectrophotometer. Invasion assays were performed similarly, with the exception that upper chambers were pre-coated with Matrigel (BD Biosciences, diluted 1:8 in serum-free medium) and polymerized for 1 h at 37 °C prior to cell seeding.

### 2.20. Flow Cytometry for Apoptosis Detection

Cell apoptosis was assessed by flow cytometry using an Annexin V-FITC/PI Apoptosis Detection Kit (BD Biosciences, Franklin Lakes, NJ, USA), as per the manufacturer’s protocol. The resulting data were analyzed with FlowJo software (Version 10.8.1, FlowJo LLC, Ashland, OR, USA).

### 2.21. In Vivo Tumorigenicity Assay

Animal procedures were approved by the Animal Ethics Committee of Northeast Normal University (NENU/IACUC, AP20240312) and conducted in compliance with institutional and national guidelines for the care and use of laboratory animals. Female athymic BALB/c nu/nu mice (5 weeks old, 16–17 g) were acquired from Charles River (Beijing, China) (*n* = 6 mice in total). To minimize inter-individual variability, a paired experimental design was employed. Specifically, HT-29 cells transfected with non-targeting control shRNA (shNC) and shTDGF1 (2 × 10^7^ cells in 200 μL sterile PBS) were subcutaneously inoculated into the left and right flanks of the same mouse, respectively (yielding 6 control tumors and 6 knockdown tumors in total). Tumor volumes were monitored every 3 days using digital calipers by an independent investigator who was blinded to the group injection assignments, and calculated as V = L × W^2^ × 0.5, where L represents the longest diameter and W the perpendicular width. After 21 days, mice were euthanized by cervical dislocation under anesthesia. The obtained tumors were weighed using a precision electronic balance. To properly account for intra-animal clustering, differences in tumor volume and weight between the shNC and shTDGF1 groups were statistically analyzed using the Two-way Repeated Measures ANOVA or paired Student’s *t*-test.

### 2.22. Immunohistochemistry (IHC)

Xenograft tumors harvested from in vivo experiments were rinsed with PBS and immersion-fixed in 10% neutral buffered paraformaldehyde. Tissues were dehydrated through a graded ethanol series (80%, 85%, 95%, and 100%), paraffin-embedded, and sectioned at 5 μm thickness onto poly-lysine-coated slides. After deparaffinization in xylene and rehydration through graded alcohols, antigen retrieval was performed in citrate buffer (pH 6.0) using microwave heating. Sections were immunostained with anti-Ki67 antibody (ZSGB-BIO, Beijing, China) according to the manufacturer’s protocol, with 3,3′-diaminobenzidine (DAB) as chromogen and hematoxylin counterstaining. Furthermore, the tissue microarray was obtained from Outdo BioTech (Shanghai, China), containing 93 COAD samples and 87 adjacent normal tissue samples with matched clinical data, including patient survival, tumor volume, gender, age, pathologic stage, TNM stage and MLH1/MSH2/MSH6/PMS2/PDL1 expression. IHC treatment was performed as previously described, following dewaxing and rehydration with anti-TDGF1. Expression levels of the markers were analyzed using ImageJ software (version 1.54d).

### 2.23. Statistical Analysis

All experimental data were expressed as mean ± standard deviation (SD). Prior to hypothesis testing, data distribution assumptions were systematically evaluated. The Shapiro–Wilk test was utilized to assess data normality, and Levene’s test was used to verify the homogeneity of variances. For normally distributed data with equal variance, statistical significance between two independent groups was assessed by the unpaired Student’s *t*-test, while multiple comparisons were performed by One-way ANOVA. Paired samples were evaluated using the paired Student’s *t*-test. For the paired in vivo tumorigenicity assay, differences in tumor growth curves measured at multiple time points were evaluated using a Two-way Repeated Measures ANOVA. Correlations were analyzed using the Pearson or Spearman method, depending on data normality. Crucially, to control for false positives in high-throughput computational analyses, the Benjamini–Hochberg (BH) method was applied to adjust *p*-values for multiple testing, and the resulting False Discovery Rate (FDR) < 0.05 or < 0.01 was defined as the significance threshold for these large-scale screenings. Survival differences for TDGF1 and ncRNAs were compared via the log-rank test. Furthermore, univariate and multivariate Cox regression analyses were utilized to identify the proper terms to build the nomogram, and hazard ratios (HRs), 95% confidence intervals (CIs), and *p*-values were visualized. The association between TDGF1 and important clinical indicators was analyzed by Student’s *t*-test or Chi-square test. Statistical significance was defined as a two-tailed *p* < 0.05 or log-rank *p* < 0.05.

## 3. Results

### 3.1. TDGF1 Exhibits Distinct, Tumor-Specific Expression Patterns Independent of Pathological Stage

To explore potential roles of TDGF1 in tumorigenesis and development, we first analyzed its expression patterns across human cancers and paired normal tissues. Compared to normal samples, TDGF1 expression was significantly upregulated in 3 cancer types (COAD, LIHC, and READ) and downregulated in 12 types (BLCA, BRCA, CHOL, GBM, HNSC, KICH, KIRC, KIRP, LUAD, LUSC, PRAD, and THCA). However, no significant differences were observed in CESC, ESCA, PAAD, PCPG, STAD or UCEC (Figure S1A). We also validated these findings using the GEPIA database. As presented in Figure S1B–P, TDGF1 expression was significantly increased in COAD and READ compared to normal controls, while obviously decreased expression was confirmed in KICH, KIRC, and KIRP. Analysis of TDGF1 levels across diverse human cancer tissues revealed higher expression in COAD, READ, TGCT, and THYM, and lower expression in KICH, KIRC, KIRP, and OV (Figure S1Q). Collectively, these results demonstrate consistent upregulation of TDGF1 in COAD and READ, and downregulation in KICH, KIRC, and KIRP. Further analysis using the TISIDB database revealed significant differences in TDGF1 expression across subtypes of COAD and READ (Figure S1R,S). However, GEPIA analysis showed no significant correlation between TDGF1 expression and pathological stage in COAD, READ, KICH, KIRC, or KIRP (Figure S1T–X). These findings together indicate that TDGF1 may function as a crucial regulator in the carcinogenesis of these five cancer types (COAD, READ, KICH, KIRC, KIRP).

### 3.2. TDGF1 Expression Is Associated with Poor Disease-Free Survival in Colon Cancer

**Figure 1:**
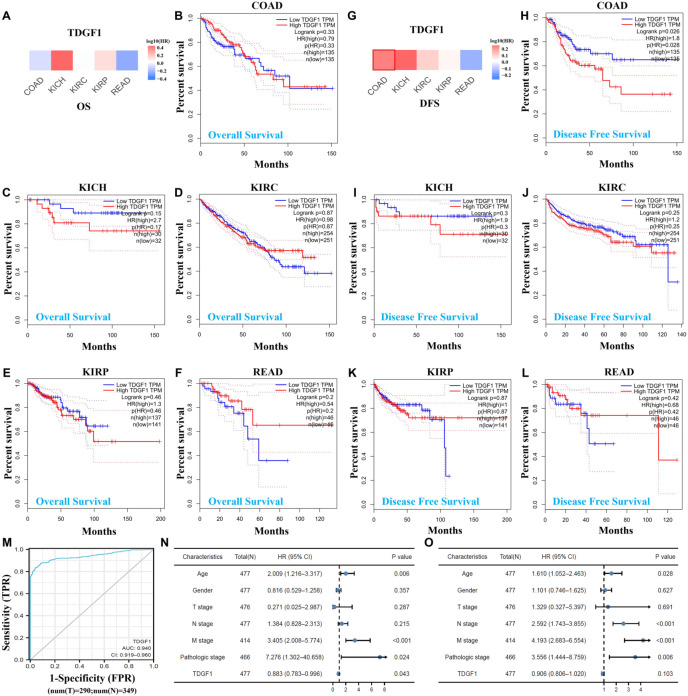
Prognostic significance of TDGF1 across malignancies via GEPIA analysis. (**A**–**F**) Heatmap showing the correlation between TDGF1 expression and the OS analyzed via the GEPIA database. The color scale indicates the correlation strength (red: positive, blue: negative). Kaplan–Meier curves were plotted for OS of patients with COAD, KICH, KIRC, KIRP, and READ stratified by high (red) and low (blue) TDGF1 expression. The dashed lines in the survival curves represent the 95% confidence intervals, log-rank *p*-values were indicated. (**G**–**L**) Heatmap showing the correlation between TDGF1 expression and the DFS analyzed via the GEPIA database. The color scale indicates the correlation strength (red: positive, blue: negative). Kaplan–Meier curves were plotted for DFS of patients with COAD, KICH, KIRC, KIRP, and READ stratified by high (red) and low (blue) TDGF1 expression. The dashed lines in the survival curves represent the 95% confidence intervals, log-rank *p*-values were indicated. (**M**) ROC curve illustrating the diagnostic performance of TDGF1 for discriminating tumor from normal tissue. AUC with 95% confidence interval was shown. (**N**,**O**) Forest plots of univariate (**N**) and multivariate (**O**) Cox proportional hazards regression analyses evaluating TDGF1 as an independent prognostic factor. Hazard ratios (HR), 95% CIs, and *p*-values were displayed.

We next evaluated the prognostic significance of TDGF1 in COAD, KICH, KIRC, KIRP, and READ using overall survival (OS) and disease-free survival (DFS). TDGF1 expression showed no significant association with OS in any cancer type (Figure 1A–F). For DFS, high TDGF1 expression was significantly associated with poor prognosis exclusively in COAD (Figure 1G–L). In addition, TDGF1 demonstrated high statistical discrimination (AUC = 0.940) for distinguishing tumors from normal tissue within the dataset (Figure 1M). Furthermore, univariate and multivariate Cox regression confirmed TDGF1 as significantly associated with colon cancer prognosis, though not as an independent prognostic factor (Figure 1N,O). These results suggest TDGF1 may serve as an unfavorable prognostic biomarker in colon cancer.

### 3.3. Hsa-miR-3614-5p Targets TDGF1 and Serves as a Favorable Prognostic Indicator in Colon Cancer

**Figure 2:**
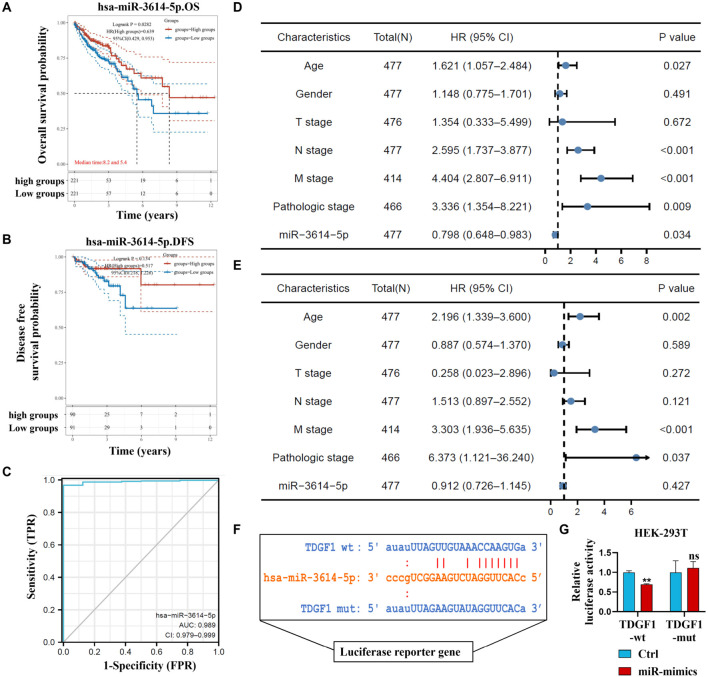
Identification of hsa-miR-3614-5p as a TDGF1-targeting miRNA with favorable prognostic value in colon cancer. (**A**,**B**) Kaplan–Meier curves for OS and DFS of COAD patients stratified by high (red) and low (blue) expression of hsa-miR-3614-5p. The dashed lines in the survival curves represent the 95% confidence intervals, log-rank *p*-values were indicated. (**C**) ROC curve analysis of hsa-miR-3614-5p for COAD classification. AUC with 95% CI was shown. (**D**,**E**) Forest plots of univariate (**D**) and multivariate (**E**) Cox proportional hazards regression analyses for hsa-miR-3614-5p in COAD. HR, 95% CIs, and *p*-values were provided. (**F**) Predicted hsa-miR-3614-5p binding sites within the 3′UTR of TDGF1 and construction of OLMALINC luciferase reporter vector. WT, wild type; Mut, mutation type. (**G**) Luciferase reporter assay validating the interaction between hsa-miR-3614-5p and the wild-type (TDGF1-wt) or mutant (TDGF1-mut) 3′UTR of TDGF1. Data presented as mean ± SD (*n* = 3). Significance was calculated with Student’s *t* test. ** *p* < 0.01, ns, no significance.

Given the established role of miRNAs in gene regulation, we employed multiple databases to predict miRNAs potentially targeting TDGF1, identifying 132 candidates (Figure S2A,B). Based on the canonical negative regulatory relationship between miRNAs and their targets, we examined expression correlations between candidate miRNAs and TDGF1 in colon cancer. As listed in Table S2, TDGF1 showed significant negative correlations (FDR < 0.01) with hsa-let-7i-5p and hsa-miR-3614-5p by Pearson’s correlation analysis. We further determined the expression patterns of these miRNAs in colon cancer. As presented in Figure S2C,D, hsa-miR-3614-5p was markedly downregulated. In terms of prognostic significance, elevated hsa-miR-3614-5p expression correlated well with improved patient prognosis (Figure 2A,B). Notably, hsa-miR-3614-5p demonstrated a strong discriminative value (AUC = 0.989) for distinguishing tumors from normal tissue in this cohort (Figure 2C). Furthermore, univariate and multivariate Cox regression confirmed its significant association with colon cancer prognosis; however, it could not be used as an independent prognostic factor (Figure 2D,E). Furthermore, hsa-miR-3614-5p binding sites were predicted in the 3′UTR of TDGF1 (Figure 2F). Luciferase assay uncovered that up-regulation of hsa-miR-3614-5p significantly attenuated the luciferase activity of pmirGLO-TDGF1-wt while showing no obvious effect on pmirGLO-TDGF1-mut (Figure 2G), indicating that TDGF1 is indeed a target of hsa-miR-3614-5p.

### 3.4. OLMALINC Functions as a Competing Endogenous RNA (ceRNA) by Targeting hsa-miR-3614-5p, and Its Elevated Expression Is Associated with Poor Outcome

**Figure 3:**
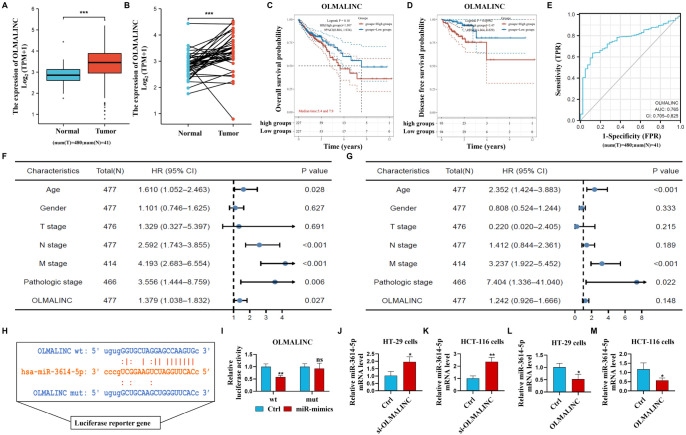
Identification of OLMALINC as a hsa-miR-3614-5p-targeting ceRNA linked to poor survival outcomes. (**A**,**B**) Expression levels of OLMALINC in unpaired (**A**) and paired (**B**) COAD tumor/normal specimens from the TCGA dataset. Data presented as mean ± SD. Significance was calculated with Student’s *t* test. *** *p* < 0.001. (**C**,**D**) Kaplan–Meier curves for OS and DFS of COAD patients stratified by high (red) and low (blue) expression of OLMALINC. The dashed lines in the survival curves represent the 95% confidence intervals, log-rank *p*-values were indicated. (**E**) ROC curve evaluating the diagnostic performance of OLMALINC for COAD classification. AUC and 95% CI were displayed. (**F**,**G**) Forest plots of univariate (**F**) and multivariate (**G**) Cox regression analyses evaluating OLMALINC as prognostic factors in COAD. HR, 95% CIs, and *p*-values were provided. (**H**) Prediction of hsa-miR-3614-5p binding sites within OLMALINC and construction of OLMALINC luciferase reporter vector. WT, wild type; Mut, mutation type. (**I**) Luciferase reporter assay in HEK-293T cells co-transfected with wild-type (OLMALINC-wt) or mutant (OLMALINC-mut) reporter plasmids and hsa-miR-3614-5p mimics or negative control (Ctrl). Renilla luciferase was used for normalization. Data presented as mean ± SD of three independent experiments (*n* = 3). Significance was calculated with Student’s *t* test. *** *p* < 0.001, ns, no significance. (**J**,**K**) Relative expression of hsa-miR-3614-5p in HT-29 (**J**) and HCT-116 (**K**) cells after transfection with siRNA targeting OLMALINC (si-OLMALINC) or negative control (Ctrl) for 48 h. Data normalized to U6 and presented as mean ± SD (*n* = 3). Significance was calculated with Student’s *t* test. * *p* < 0.05, ** *p* < 0.01. (**L**,**M**) Relative expression of hsa-miR-3614-5p in HT-29 (**L**) and HCT-116 (**M**) cells after transfection with OLMALINC overexpression plasmid (OLMALINC) or empty vector (Ctrl) for 48 h. Data normalized to U6 and presented as mean ± SD (*n* = 3). Significance was calculated with Student’s *t* test. * *p* < 0.05.

Using ENCORI, we identified 32 potential upstream lncRNAs of hsa-miR-3614-5p (Figure S3A). Based on the ceRNA mechanism, lncRNAs elevate mRNA expression by competitively binding shared miRNAs, implying: (i) negative lncRNA-miRNA correlation, and (ii) positive lncRNA-mRNA correlation. In colon cancer, LINC01089 and OLMALINC showed significant negative correlation with hsa-miR-3614-5p and positive correlation with TDGF1 (Spearman correlation, FDR < 0.05) (Figure S3B–E, Tables S3 and S4). Meanwhile, both lncRNAs were significantly upregulated in tumor versus normal tissue (Figure 3A,B and Figure S3F,G). Prognostic analysis revealed OLMALINC (but not LINC01089) could serve as a non-independent factor affecting the prognosis of patients with colon cancer, with promising diagnostic value for tumor discrimination (AUC = 0.765) (Figure 3C–G and Figure S3H,I). The subcellular localization of lncRNA is closely associated with its biological function. We conducted cellular fractionation assays and found that OLMALINC was enriched in the cytoplasmic fraction in HT-29 and HCT-116 cells (Figure S4A,B). To validate the direct interaction between hsa-miR-3614-5p and OLMALINC, dual-luciferase reporter assays were performed. Bioinformatics analysis predicted complementary sequences between hsa-miR-3614-5p and wild-type (wt) OLMALINC, with mutant (mut) constructs serving as controls (Figure 3H). Luciferase assay showed that up-regulation of hsa-miR-3614-5p significantly suppressed the luciferase activity in pmirGLO-OLMALINC-wt transfectants but not in mut controls, indicating that hsa-miR-3614-5p could directly bind to OLMALINC through the binding sites (Figure 3I). Subsequent functional studies revealed that OLMALINC silencing in HT-29 and HCT-116 cells significantly elevated hsa-miR-3614-5p levels (Figure 3J,K), whereas OLMALINC overexpression noticeably decreased the levels of hsa-miR-3614-5p (Figure 3L,M). Collectively, these data reveal that hsa-miR-3614-5p serves as the primary mediator of OLMALINC’s functional effects.

### 3.5. OLMALINC Regulates TDGF1 via hsa-miR-3614-5p

**Figure 4:**
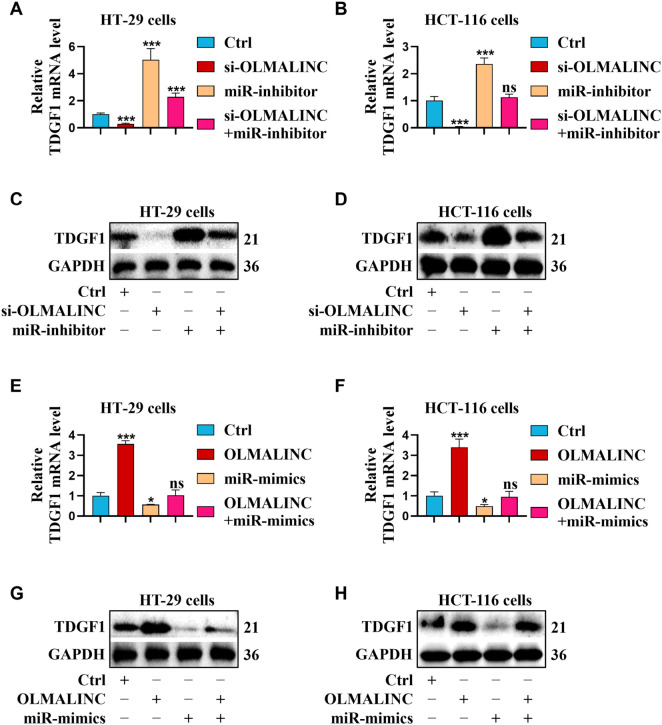
TDGF1 expression is regulated by OLMALINC via hsa-miR-3614-5p sponging. (**A**–**D**) TDGF1 mRNA (**A**,**B**) and protein (**C**,**D**) levels were measured in HT-29 and HCT-116 cells after transfection with si-OLMALINC alone or together with hsa-miR-3614-5p inhibitor. GAPDH served as loading control. Data presented as mean ± SD (*n* = 3). Significance was calculated with one-way ANOVA. *** *p* < 0.001, ns, no significance vs. Ctrl group. (**E**–**H**) TDGF1 mRNA (**E**,**F**) and protein (**G**,**H**) levels were measured in HT-29 and HCT-116 cells after transfection with OLMALINC alone or together with hsa-miR-3614-5p mimics. GAPDH served as loading control. Data presented as mean ± SD (*n* = 3). Significance was calculated with one-way ANOVA. * *p* < 0.05, *** *p* < 0.001, ns, no significance vs. Ctrl group.

We next investigated whether OLMALINC affects the target gene TDGF1 through its interaction with hsa-miR-3614-5p. In HT-29 and HCT-116 cells, we identified that OLMALINC knockdown led to a significant decrease in TDGF1 at both mRNA and protein levels (Figure 4A–D). However, hsa-miR-3614-5p inhibition dramatically increased TDGF1 levels (Figure 4A–D). Furthermore, co-knockdown of OLMALINC and hsa-miR-3614-5p partially rescued TDGF1 suppression (vs. OLMALINC inhibition alone; Figure 4A–D). Conversely, overexpression of OLMALINC upregulated TDGF1, while hsa-miR-3614-5p mimics reduced its expression dramatically compared with the control group (Figure 4E–H). Concurrent overexpression of OLMALINC and hsa-miR-3614-5p abrogated these effects (Figure 4E–H). Taken together, these data establish a ceRNA network wherein OLMALINC sequesters hsa-miR-3614-5p to derepress TDGF1 expression, thereby affecting colon cancer progression.

### 3.6. The hsa-miR-3614-5p Overexpression or TDGF1 Suppression Reverses the Malignant Progression of Colon Cancer Driven by OLMALINC

**Figure 5:**
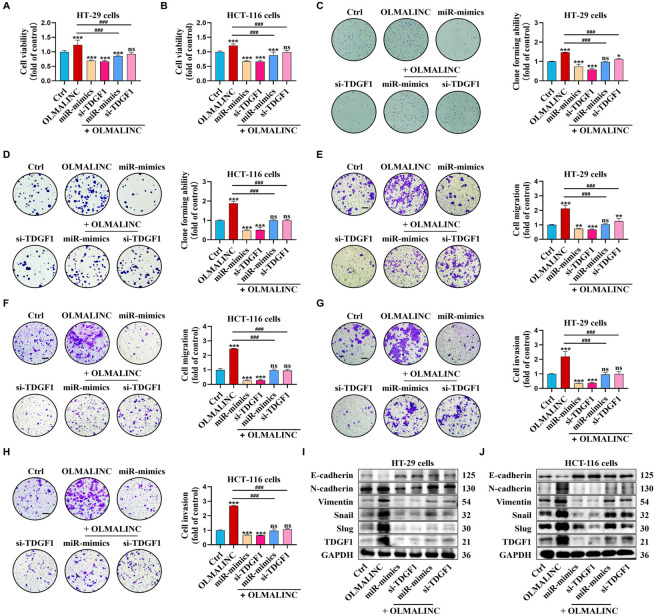
TDGF1 serves as a mediator for the OLMALINC/hsa-miR-3614-5p axis in regulating colon cell proliferation and motility. (**A**–**J**) Inhibition experiments showing that up-regulation of hsa-miR-3614-5p or knockdown of TDGF1 counteracted the promotive effects of OLMALINC overexpression on cell proliferation (**A**,**B**), colony formation (**C**,**D**), migration (**E**,**F**), invasion (**G**,**H**), and EMT protein levels (**I**,**J**). GAPDH served as loading control. Data presented as mean ± SD from three independent experiments (*n* = 3). Significance was calculated using one-way ANOVA. ns, no significance, * *p* < 0.05, ** *p* < 0.01, *** *p* < 0.001 vs. Ctrl group; ^###^ *p* < 0.001 vs. OLMALINC group.

To validate the functional relevance of the OLMALINC/hsa-miR-3614-5p/TDGF1 regulatory axis, we performed a series of rescue experiments in HT-29 and HCT-116 colon cancer cell lines. The qRT-PCR verified the successful overexpression of both OLMALINC and hsa-miR-3614-5p in the co-transfected groups of the rescue experiment (Figure S5A–D). As expected, OLMALINC overexpression robustly enhanced multiple malignant phenotypes in both cell lines. However, co-transfection with the hsa-miR-3614-5p mimics or TDGF1 siRNA significantly counteracted these pro-tumorigenic effects. Specifically, TDGF1 silencing restored the accelerated proliferation rate to baseline levels (si-TDGF1 + OLMALINC vs. Ctrl, *p* = 0.1391 in HT-29 cells and *p* = 0.7669 in HCT-116 cells). In contrast, co-transfection with hsa-miR-3614-5p mimics not only neutralized the OLMALINC-driven enhancement but also led to a significant decrease below the control levels (miR-mimics + OLMALINC vs. Ctrl, *p* = 0.0055 in HT-29 cells and *p* = 0.0067 in HCT-116 cells) (Figure 5A,B). Similarly, the enhanced clonogenic ability was reversed to normal levels by both interventions in HCT-116 cells (miR-mimics + OLMALINC vs. Ctrl, *p* = 0.8665; si-TDGF1 + OLMALINC vs. Ctrl, *p* = 0.9816), and by hsa-miR-3614-5p mimics in HT-29 cells (miR-mimics + OLMALINC vs. Ctrl, *p* = 0.6675). However, TDGF1 silencing resulted in only a partial rescue of colony formation in HT-29 cells (si-TDGF1 + OLMALINC vs. Ctrl, *p* = 0.0159) (Figure 5C,D). Regarding metastatic potential, both hsa-miR-3614-5p mimics and si-TDGF1 blunted the OLMALINC-induced migration to normal control levels in HCT-116 cells (miR-mimics + OLMALINC vs. Ctrl, *p* = 0.6414; si-TDGF1 + OLMALINC vs. Ctrl, *p* = 0.0628). In HT-29 cells, hsa-miR-3614-5p overexpression restored migratory capacity to baseline (miR-mimics + OLMALINC vs. Ctrl, *p* = 0.5736), whereas TDGF1 knockdown partially rescued the migratory phenotype (si-TDGF1 + OLMALINC vs. Ctrl, *p* = 0.0009) (Figure 5E,F). Unlike migration, the OLMALINC-driven invasive capacity was reversed to normal levels by both hsa-miR-3614-5p mimics and TDGF1 siRNA in both cell lines (HT-29: miR-mimics + OLMALINC vs. Ctrl, *p* = 0.3872; si-TDGF1 + OLMALINC vs. Ctrl, *p* = 0.6228. HCT-116: miR-mimics + OLMALINC vs. Ctrl, *p* = 0.5289; si-TDGF1 + OLMALINC vs. Ctrl, *p* = 0.1751) (Figure 5G,H). At the molecular level, these interventions also reversed the EMT marker expression pattern induced by OLMALINC overexpression (Figure 5I,J). Collectively, these data demonstrate that OLMALINC promotes colon cancer progression, at least in part, through the hsa-miR-3614-5p/TDGF1 axis.

### 3.7. Functional Rescue Assays in OLMALINC-Knockdown Cells Verify the hsa-miR-3614-5p/TDGF1 Axis as a Downstream Effector

**Figure 6:**
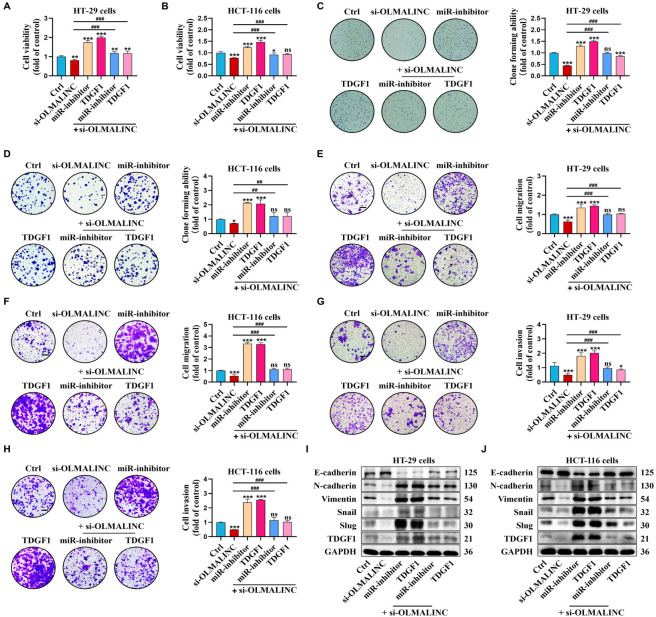
TDGF1 mediates the effects of OLMALINC/hsa-miR-3614-5p axis on colon cell proliferation and motility. (**A**–**J**) Rescue experiments showing that down-regulation of hsa-miR-3614-5p or overexpression of TDGF1 reversed the suppressive effects of OLMALINC knockdown on cell proliferation (**A**,**B**), colony formation (**C**,**D**), migration (**E**,**F**), invasion (**G**,**H**), and EMT protein levels (**I**,**J**). GAPDH served as loading control. Data presented as mean ± SD from three independent experiments (*n* = 3). Significance was calculated using one-way ANOVA. ns, no significance, * *p* < 0.05, ** *p* < 0.01, and *** *p* < 0.001 vs. Ctrl group; ^##^ *p* < 0.01 and ^###^ *p* < 0.001 vs. si-OLMALINC group.

To further validate the above regulatory relationship and provide bidirectional mechanistic evidence, we performed reverse rescue experiments. The qRT-PCR verified the successful inhibition of both OLMALINC and hsa-miR-3614-5p in the co-transfected groups of the rescue experiment (Figure S5E–H). In stark contrast to the phenotypic promotion observed in the overexpression assays, siRNA-mediated OLMALINC silencing significantly impaired multiple malignant phenotypes. Importantly, these suppressive effects were significantly attenuated by either the inhibition of hsa-miR-3614-5p or the ectopic overexpression of TDGF1. Specifically, in HT-29 cells, both hsa-miR-3614-5p inhibition and TDGF1 overexpression robustly reversed the diminished proliferation; notably, these interventions further enhanced the cell viability to levels significantly higher than the control group (miR-inhibitor + si-OLMALINC vs. Ctrl, *p* = 0.0016; TDGF1 + si-OLMALINC vs. Ctrl, *p* = 0.0018). Conversely, in HCT-116 cells, TDGF1 overexpression restored the impaired cell viability to normal baseline levels (TDGF1 + si-OLMALINC vs. Ctrl, *p* = 0.1118), whereas the hsa-miR-3614-5p inhibitor provided a partial rescue, with viability remaining significantly lower than the control group (miR-inhibitor + si-OLMALINC vs. Ctrl, *p* = 0.0243) (Figure 6A,B). Meanwhile, both interventions rescued the clonogenic capacity to normal levels in HCT-116 cells (miR-inhibitor + si-OLMALINC vs. Ctrl, *p* = 0.1759; TDGF1 + si-OLMALINC vs. Ctrl, *p* = 0.1575). In HT-29 cells, the miR-3614-5p inhibitor restored this ability (miR-inhibitor + si-OLMALINC vs. Ctrl, *p* = 0.7464), while TDGF1 overexpression provided a partial rescue (TDGF1 + si-OLMALINC vs. Ctrl, *p* = 0.0002) (Figure 6C,D). For cell migration, the impaired transwell migratory potentials driven by OLMALINC silencing were rescued to normal baseline levels by both the miR-3614-5p inhibitor and TDGF1 overexpression across both HT-29 and HCT-116 cell lines (HT-29: miR-inhibitor + si-OLMALINC vs. Ctrl, *p* = 0.6140; TDGF1 + si-OLMALINC vs. Ctrl, *p* = 0.3136. HCT-116: miR-inhibitor + si-OLMALINC vs. Ctrl, *p* = 0.2449; TDGF1 + si-OLMALINC vs. Ctrl, *p* = 0.1474) (Figure 6E,F). Similar to migration, restoration of invasive capacity to control levels was observed in HCT-116 cells for both interventions (miR-inhibitor + si-OLMALINC vs. Ctrl, *p* = 0.2236; TDGF1 + si-OLMALINC vs. Ctrl, *p* = 0.7596), as well as in the miR-inhibitor-treated HT-29 cells (miR-inhibitor + si-OLMALINC vs. Ctrl, *p* = 0.1353). However, TDGF1 overexpression resulted in a partial rescue of invasion in HT-29 cells (TDGF1 + si-OLMALINC vs. Ctrl, *p* = 0.0311) (Figure 6G,H). With respect to molecular changes, both treatment strategies counteracted the suppressive effect of OLMALINC silencing on the EMT-related expression signature (Figure 6I,J). These reciprocal rescue results provide compelling bidirectional evidence that OLMALINC exerts its oncogenic functions in colon cancer by sponging hsa-miR-3614-5p to de-repress TDGF1.

### 3.8. TDGF1 Facilitates the Malignant Progression of Colon Cancer Cells, and Thr88 Mutation Partially Impairs Its Oncogenic Function

Emerging evidence indicates that TDGF1 critically regulates cell proliferation, motility, and survival through dual signaling mechanisms: a Glypican-1-mediated activation of the MAPK/AKT pathway and a Nodal-dependent Smad pathway [38,39]. While a key post-translational modification, O-fucosylation at the conserved threonine residue (Thr88 in human TDGF1) within its EGF-like domain, is essential for its co-receptor function in facilitating Nodal signaling [40,41,42]. To elucidate the specific role of TDGF1 and its O-fucosylation in colorectal cancer pathogenesis, we expressed wild-type human TDGF1 and the T88A mutant in human colon cancer cell lines.

**Figure 7:**
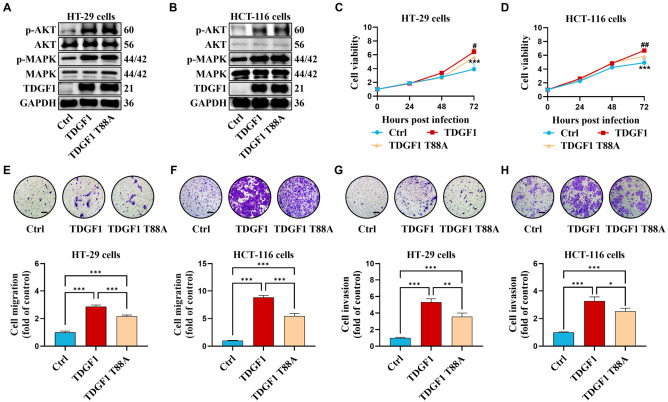
TDGF1 promotes the malignant progression of colon cancer cells, an effect that is partially attenuated by the Thr88 mutation. (**A**,**B**) Western blot analysis of MAPK/AKT signaling proteins in (**A**) HT-29 and (**B**) HCT-116 cells transfected with control vector (Ctrl), wild-type TDGF1 (TDGF1), or the TDGF1 T88A mutant (TDGF1 T88A). GAPDH served as loading control (*n* = 3). (**C**,**D**) Cell viability of (**C**) HT-29 and (**D**) HCT-116 cells overexpressing TDGF1 or TDGF1 T88A, measured by MTT assay over 72 h. Data presented as mean ± SD (*n* = 3). Significance was calculated with one-way ANOVA. *** *p* < 0.001 vs. TDGF1 or TDGF1 T88A group; ^#^ *p* < 0.05, ^##^ *p* < 0.01 vs. TDGF1 T88A group at the same time point. (**E**–**H**) The migration (**E**,**F**) and invasion (**G**,**H**) abilities of HT-29 and HCT-116 cells expressing TDGF1 or TDGF1 T88A were assessed by Transwell assays without or with Matrigel, respectively. Migrated and invaded cells were quantified and presented as fold change relative to the Ctrl group (mean ± SD, *n* = 3). Significance was calculated with one-way ANOVA. * *p* < 0.05, ** *p* < 0.01, and *** *p* < 0.001.

First, we examined the effect of TDGF1 on the oncogenic phenotypes of colon cancer cells. Western blot analyses in HT-29 and HCT-116 cells demonstrated that overexpression of both wild-type TDGF1 and the T88A mutant robustly elevated the phosphorylation of AKT and MAPK to a similar extent, while the total protein levels of AKT and MAPK remained unaltered (Figure 7A,B). Further functional assays, including cell viability, transwell migration, and invasion, revealed that TDGF1 overexpression significantly accelerated the proliferation of HT-29 and HCT-116 cells, and markedly augmented their motility and invasive capacity. While the TDGF1 T88A mutant exhibited a profound loss of function compared to the wild-type protein, it still conferred enhanced malignant phenotypes to colon cancer cells relative to the control group (Figure 7C–H). These data suggest that TDGF1 drives colon cancer progression, and while the Thr88 residue is critical for its full oncogenic activity, it may also exert effects through Thr88-independent mechanisms.

### 3.9. TDGF1 Mediates Dual Signaling Activation Through Thr88-Dependent Nodal/Smad2 and Glypican-1/MAPK-AKT Axes

**Figure 8:**
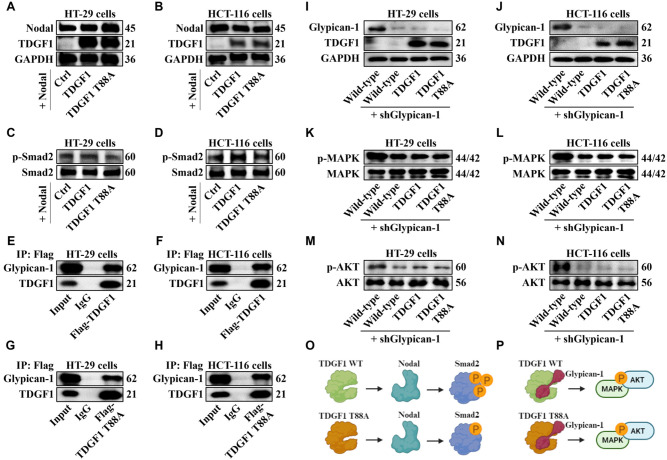
TDGF1 drives dual signaling activation by engaging the Thr88-dependent Nodal/Smad2 axis together with the Glypican-1/MAPK-AKT axis. (**A**–**D**) Nodal, TDGF1, and p-Smad2 levels were detected in HT-29 and HCT-116 cells co-transfected with a Nodal plasmid and either control vector, TDGF1, or TDGF1 T88A. GAPDH or Smad2 served as loading control. *N* = 3 biological replication. (**E**–**H**) Co-immunoprecipitation assays validating the interaction between TDGF1 or TDGF1 T88A and Glypican-1. HT-29 and HCT-116 cells were transfected with Flag-tagged TDGF1-WT or TDGF1-T88A. Cell lysates were immunoprecipitated with an anti-Flag antibody, followed by immunoblotting with anti-Glypican-1 and anti-Flag antibodies. Input lysates confirm the expression of target proteins. *N* = 3 biological replication. (**I**–**N**) Western blot analysis was performed to investigate the impact of Glypican-1 knockdown on TDGF1- or TDGF1T88A-mediated activation of MAPK/AKT signaling. Specifically, expression levels of Glypican-1, TDGF1, p-MAPK, and p-AKT were assessed in HT-29 and HCT-116 cells transfected with either Glypican-1-targeting shRNA (shGPC1) or control shRNA (shNC), in combination with overexpression of empty vector, TDGF1-WT, or TDGF1-T88A. GAPDH, MAPK, or AKT served as loading control. *N* = 3 biological replication. (**O**,**P**) Schematic diagram of TDGF1 and the TDGF1 T88A mutant activating the Nodal/Smad2 pathway and the Glypican-1-dependent MAPK/AKT signaling cascade.

To dissect the dual signaling axes downstream of TDGF1, we separately examined the Nodal-Smad2 pathway and the Glypican-1-MAPK/AKT pathway. Whether the T88A mutant could cooperate with Nodal to activate Smad2 in colon cancer cells was investigated. Following co-transfection of flag-tagged TDGF1 or flag-tagged TDGF1 T88A along with myc-tagged Nodal into HT-29 and HCT-116 cells, Western blot analysis confirmed successful overexpression of TDGF1/Nodal and TDGF1 T88A/Nodal in both cell lines (Figure 8A,B). Exogenous TDGF1 further enhanced Smad2 phosphorylation in Nodal-expressing cells; in contrast, Smad2 phosphorylation was markedly reduced in cells expressing the TDGF1 T88A mutant in the presence of Nodal (Figure 8C,D). These results indicate that the Thr88 residue of TDGF1 is required for full activation of the Nodal/Smad2 signaling axis in colon cancer cells.

Given that TDGF1 has been reported to activate a Nodal-independent signaling pathway via Glypican-1 [39], we next examined whether the T88A mutant retains the ability to interact with Glypican-1. Co-immunoprecipitation assays performed in HT-29 and HCT-116 cells revealed that both wild-type and T88A mutant TDGF1 proteins bound to Glypican-1 to a similar extent (Figure 8E–H). Western blot analysis of cell lysates confirmed expression of the transfected constructs and endogenous Glypican-1 (Figure 8E–H). To determine whether Glypican-1 is required for TDGF1-mediated activation of downstream MAPK/AKT signaling, wild-type cells, TDGF1-overexpressing cells, and T88A-expressing cells were transfected with either a Glypican-1-specific shRNA or a non-silencing control shRNA. Compared with control shRNA-transfected cells, Glypican-1 shRNA strongly reduced endogenous Glypican-1 expression in all three cell types (Figure 8I,J). Moreover, phosphorylation levels of MAPK (Figure 8K,L) and AKT (Figure 8M,N) were markedly decreased in Glypican-1 knockdown cells relative to controls, indicating that TDGF1-mediated activation of MAPK/AKT signaling depends on Glypican-1 but not on the Thr88 residue. Collectively, these results demonstrate that TDGF1 promotes colon cancer progression through the co-activation of two distinct signaling pathways, including the Thr88-dependent Nodal/Smad2 pathway and the Glypican-1-dependent MAPK/AKT signaling axis (Figure 8O,P).

### 3.10. TDGF1 Correlates with Immune Infiltration and Cell–Cell Communication, and Regulates Apoptosis in Colon Cancer

**Figure 9:**
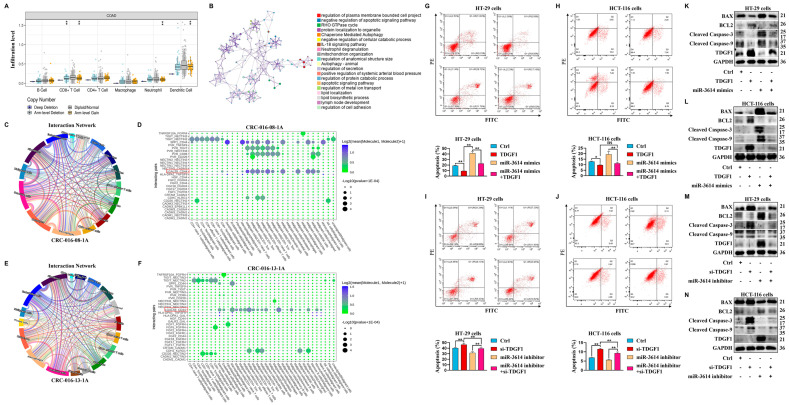
TDGF1 exerts regulatory effects in colon cancer by influencing immune infiltration, cell–cell communication, and apoptosis. (**A**) Infiltration levels of various immune cell types in COAD samples stratified by TDGF1 copy number variation (CNV) status, analyzed via TIMER database. (**B**) Network visualization of representative enriched terms from the cluster analysis. (**C**,**D**) Cell–cell interaction networks construction for single-cell samples CRC-016-08-1A and CRC-016-13-1A. (**E**,**F**) Dot plot showing ligand-receptor pairs mediating the interactions between malignant cells and distinct cell types in CRC-016-08-1A and CRC-016-13-1A. The red box specifically highlights the LGALS9-CD44 interaction pair enriched in malignant cell-T cell communication. (**G**–**J**) Schematic of apoptosis detection by flow cytometry and quantification of apoptosis rate in HT-29 and HCT-116 cells after modulation of hsa-miR-3614-5p and TDGF1. Data presented as mean ± SD (*n* = 3). Significance was calculated with one-way ANOVA. * *p* value < 0.05, ** *p* < 0.01, and ns, no significance. (**K**–**N**) Western blot analysis of anti-apoptotic and pro-apoptotic protein levels in HT-29 and HCT-116 cells after hsa-miR-3614-5p and TDGF1 modulation. GAPDH served as loading control. Data presented as mean ± SD (*n* = 3).

Given established reports implicating TDGF1 in lymph node metastasis and invasion depth, we first assessed its impact on immune infiltration in colon cancer. Analysis of somatic copy number alterations at the TDGF1 locus revealed significant modulation of CD8^+^ T cell, neutrophil, and dendritic cell infiltration (FDR < 0.01, two-sided Wilcoxon rank-sum test) (Figure 9A). TDGF1 expression positively correlated with tumor purity but negatively correlated with infiltration levels of CD8^+^ T cells, neutrophils, and dendritic cells (FDR < 0.05, Purity adjustment, Spearman correlation) (Figure S6A–G). Comprehensive algorithm-based analyses (XCELL, CIBERSORT, MCPCOUNTER, TIMER, QUANTISEQ, TIDE, EPIC) showed that elevated TDGF1 was associated with increased infiltration of central memory CD4^+^ T cells, M0 macrophages, and M2 macrophages, while most other immune subtypes were diminished (FDR < 0.05, Purity adjustment, Spearman correlation) (Figure S6H). Consistently, TDGF1 expression exhibited significant negative correlations with biomarkers of B cells, T cell subsets, macrophages, neutrophils, and dendritic cells (FDR < 0.05, Pearson correlation) (Table 1), as well as with immune checkpoint molecules PDCD1, CD274, and CTLA4 (Purity adjustment, Spearman correlation) (Figure S6I–N).

To further explore the functional implications of TDGF1, we constructed co-expression networks using LinkedOmics. A total of 1976 positively and 1857 negatively co-expressed genes were identified (FDR < 0.01, Figure S7A,B, Table S5), and 50 experimentally validated TDGF1-binding partners were retrieved from STRING (Figure S7C). Gene Ontology and KEGG pathway analyses of the co-expressed gene set revealed significant enrichment in “regulation of adaptive immune response”, “response to molecule of bacterial origin”, “cytokine-cytokine receptor interaction”, and “apoptosis” (FDR < 0.01, Figure S7D,E). Single-cell RNA-seq analysis further characterized heterogeneous TDGF1 expression across malignant and immune cell subsets in the colorectal cancer (CRC) microenvironment (these specific single-cell datasets encompass both colon and rectal cancer samples) (Figures S8–S10). Focusing on datasets where TDGF1 was exclusively expressed in malignant cells (CRC-016-08-1A, CRC-016-12-1A, CRC-016-13-1A), we identified differentially expressed genes positively (*n* = 182) or negatively (*n* = 36) correlated with TDGF1, including NDUFAB1, TSPAN6, DPM1, M6PR, FKBP4, CFTR, ICA1, CD38 and AOC1 (FDR < 0.05, Pearson correlation) (Figure S11A,B, Table S6). Functional annotation of these TDGF1-associated genes highlighted enrichment in negative regulation of apoptotic signaling, immunoregulation, and autophagy (FDR < 0.01) (Figure 9B and Figure S11C,D, Table S7). Additionally, a protein–protein interaction network was also constructed for these differentially expressed genes (Figure S12). Using the same single-cell datasets (CRC-016-08-1A and CRC-016-13-1A), we reconstructed cell–cell communication networks, verified established interactions between malignant cells and multiple immune/stromal populations, including T cell subsets, dendritic cells, endothelial cells, fibroblasts, macrophages, monocytes, and NK cells, and identified the LGALS9-CD44 ligand-receptor pair as significantly enriched in malignant cell-T cell interactions (Figure 9C–F and Figures S13—S16). Notably, CD44 showed significant positive co-expression with TDGF1 in the single-cell analysis (Table S6), suggesting that TDGF1 may be intricately linked to tumor-immune crosstalk via the LGALS9-CD44 axis. However, direct functional validation is required to definitively establish its causal role in immune regulation.

To experimentally validate the predicted role of TDGF1 targeted by hsa-miR-3614-5p in apoptosis, we performed functional assays in colon cancer cells. As shown by flow cytometry (Figure 9G,H), hsa-miR-3614-5p mimics significantly increased the apoptotic rate, confirming its pro-apoptotic function, and ectopic TDGF1 overexpression markedly attenuated this effect. Conversely, inhibition of endogenous miR-3614-5p suppressed apoptosis, whereas siRNA-mediated TDGF1 knockdown effectively reversed this protective outcome (Figure 9I,J). Western blotting analysis (Figure 9K,L) revealed that hsa-miR-3614-5p mimics upregulated pro-apoptotic markers (BAX, cleaved Caspase-3/9) and downregulated the anti-apoptotic protein BCL2, while TDGF1 overexpression reversed this profile. Furthermore, TDGF1 knockdown enhanced the pro-apoptotic cascade even in the presence of the miR-3614-5p inhibitor (Figure 9M,N). Collectively, these integrated analyses confirm that TDGF1 contributes to colon cancer pathogenesis through an anti-apoptotic mechanism mediated by the hsa-miR-3614-5p/TDGF1 axis. In addition, bioinformatic analyses reveal that TDGF1 expression is associated with distinct immune-related pathways.

### 3.11. Knockdown of TDGF1 Suppresses Tumorigenesis in a Colon Cancer Xenograft Model

**Figure 10:**
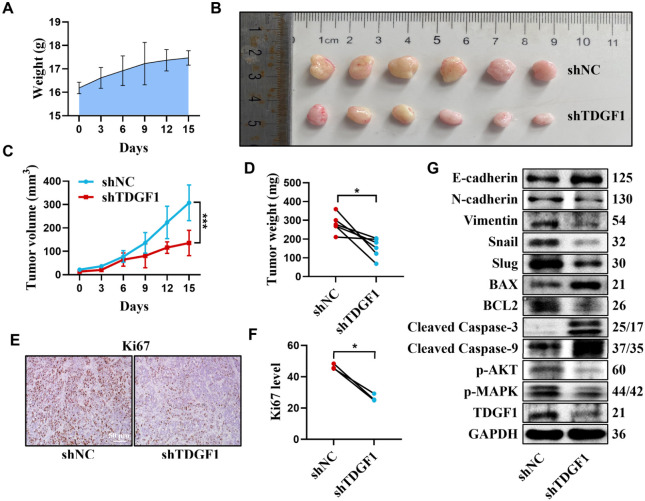
Knockdown of TDGF1 inhibits tumor growth in vivo. (**A**) Body weight curves of mice during the experiment (*n* = 6). Data were presented as mean ± SD. (**B**) Representative images of excised tumors from the paired shNC and shTDGF1 groups (*n* = 6 in each group). (**C**) Tumor growth curves plotted according to tumor volume measurements over time (*n* = 6 in each group). Significance was calculated with Two-way repeated measures ANOVA. *** *p* < 0.001. (**D**) Weights of excised tumors at the endpoint (*n* = 6 in each group). Significance was calculated with the paired Student’s *t* test. * *p* < 0.05. (**E**,**F**) Representative IHC images (**E**) and quantification (**F**) of Ki67 staining in paired tumor tissues (*n* = 3 in each group). Scale bar = 50 μm. Significance was calculated with the paired Student’s *t* test. * *p* < 0.05. (**G**) Western blot analysis of EMT and apoptosis-related proteins as well as the activation status of MAPK/AKT signaling in xenograft tumor tissues. GAPDH was used as the loading control. *N* = 3 biological replication.

To investigate the functional role of TDGF1 in colon cancer progression in vivo, we established a xenograft tumor model. The results revealed a progressive increase in murine body weight over the experimental timeline (Figure 10A). Critically, TDGF1 knockdown significantly attenuated tumor growth and related oncogenic phenotypes relative to the control group, as evidenced by reduced tumor volume, decreased tumor weight, and diminished Ki67 proliferation index, enhanced pro-apoptotic signaling (elevated BAX, Cleaved Caspase-3, and Cleaved Caspase-9), weakened anti-apoptotic signaling (reduced BCL2), and downregulated EMT and MAPK/AKT signaling levels at endpoint analyses (Figure 10B–G). These data collectively demonstrate that TDGF1 functionally promotes tumorigenesis in colon cancer through the regulation of proliferative capacity in vivo.

### 3.12. TDGF1 Is Established as a Prognostic Biomarker in Colon Cancer by Clinical Evidence, Linked to Advanced Tumor Stage and MMR Deficiency

**Figure 11:**
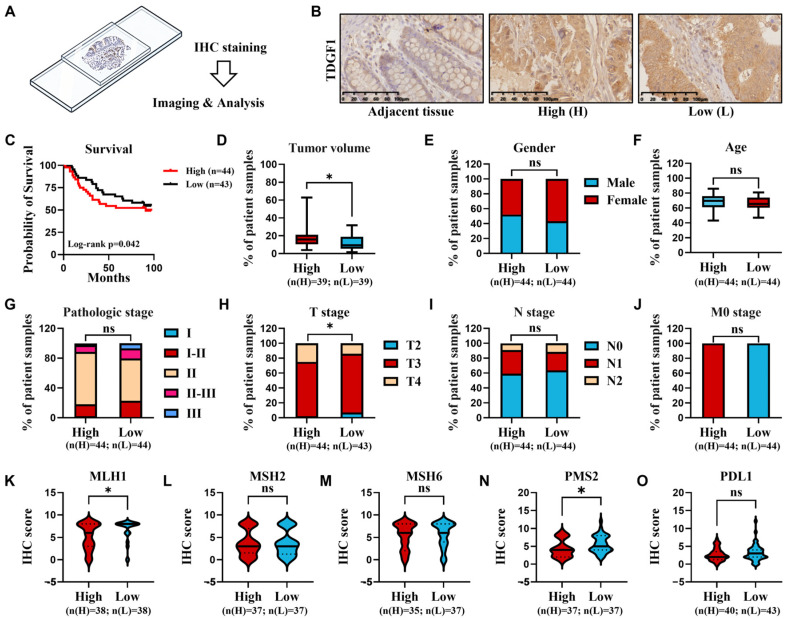
TDGF1 is associated with advanced tumor stage and impaired mismatch repair in colon cancer. (**A**) Flowchart of IHC staining for tissue microarrays. (**B**) IHC staining showing TDGF1 expression in tumor (*n* = 93) and adjacent normal tissues (*n* = 87), scale bar = 100 μm. Patients were then stratified into high (H) and low (L) TDGF1 expression groups according to staining intensity. (**C**) Kaplan–Meier survival curve for overall survival of colon cancer patients stratified by high (*n* = 44) and low (*n* = 43) TDGF1 IHC score. Log-rank *p*-value was indicated. (**D**–**O**) Correlation analysis between TDGF1 IHC score and clinicopathological parameters: tumor volume (**D**), gender (**E**), age (**F**), pathologic stage (**G**), T stage (**H**), N stage (**I**), M stage (**J**), and protein levels of MMR markers MLH1 (**K**), MSH2 (**L**), MSH6 (**M**), PMS2 (**N**), and PD-L1 (**O**). Data presented as mean ± SD. Significance was calculated with Spearman, Chi-square or Student’s *t* test. * *p* value < 0.05, and ns, no significance.

To evaluate the clinical relevance of TDGF1 in colon cancer, we performed IHC staining and quantitative image analysis on tumor tissues and adjacent normal tissues from patient cohorts (Figure 11A). Representative IHC images demonstrated significantly elevated TDGF1 expression in tumor tissues compared with adjacent non-tumor tissues, and we further stratified patients into high (H) and low (L) TDGF1 expression groups based on staining intensity (Figure 11B). Survival analysis revealed that patients with high TDGF1 expression had a significantly shorter overall survival than those with low expression (log-rank *p* = 0.042; Figure 11C). Moreover, tumors in the TDGF1-high group were 1.5-fold larger than those in the TDGF1-low group (Figure 11D). Correlation analysis with clinicopathological characteristics showed no significant association between TDGF1 expression and patient gender, age, pathologic stage, N stage, or M0 stage. However, high TDGF1 expression was significantly correlated with advanced T stage; for instance, the proportion of T4 stage tumors was higher in the TDGF1-high group (25.00%, 11/44) than in the TDGF1-low group (13.95%, 6/43) (Figure 11E–J). Given the critical role of DNA MMR proteins (MLH1, MSH2, MSH6, and PMS2) in maintaining genomic integrity and their deficiency leading to microsatellite instability (MSI), a key event in colon carcinogenesis, we assessed their relationship with TDGF1. As shown in Figure 11K–N, expression levels of MLH1 and PMS2 were significantly reduced in TDGF1-high tumors compared with TDGF1-low group, whereas no significant differences were observed for MSH2 and MSH6. However, subsequent in vitro validations demonstrated that TDGF1 knockdown did not alter the protein levels of MLH1 or PMS2 in colon cancer cells (Figure S17). We further investigated the association between TDGF1 and key immune-related markers. IHC scoring indicated a modest decrease in PDL1 level in the TDGF1-high group relative to the TDGF1-low group, although the difference was not statistically significant (Figure 11O). Collectively, these clinical findings establish TDGF1 as a possible prognostic biomarker in colon cancer, linking its overexpression to more advanced tumor progression, altered expression patterns of specific mismatch repair proteins, and unfavorable patient outcomes.

## 4. Discussion

Colon cancer is a prevalent malignancy necessitating robust prognostic biomarkers and therapeutic targets. While TDGF1 is implicated in multiple cancers, its functional network in colon cancer has remained incomprehensive. In this study, we establish TDGF1 as a critical oncogenic mediator. Our integrated analyses revealed that TDGF1 is significantly upregulated in colon cancer and correlates with poor DFS. Functional validations demonstrated that TDGF1 fundamentally drives cancer progression, as its knockdown significantly attenuated in vitro proliferation, migration, invasion, and in vivo tumorigenic capacity, whereas its overexpression promoted these oncogenic processes. These findings align with previous reports highlighting the role of TDGF1 in primary colon cancer and metastatic lymph nodes [43], as well as its targetability using anti-TDGF1 antibodies [44], firmly establishing its role as a driver of colon cancer progression.

Regarding upstream regulation, substantial evidence documents the involvement of noncoding RNAs in post-transcriptional gene regulation via competing endogenous RNA (ceRNA) networks [45,46,47,48,49], consistent with the canonical ceRNA hypothesis [50]. Instead of relying solely on expression profiling, we integrated computational screening with functional validation to delineate the upstream mechanisms governing TDGF1. We identified hsa-miR-3614-5p as the predominant tumor-suppressive miRNA targeting TDGF1, corroborating clinical evidence that links low hsa-miR-3614-5p expression to advanced tumor stages [45]. Furthermore, we pinpointed OLMALINC, which was previously implicated in tumor progression via miR-147a axes [51], as the principal oncogenic lncRNA. The dual-luciferase and functional rescue assays confirmed that OLMALINC acts as a molecular sponge for hsa-miR-3614-5p. Together, these data establish the OLMALINC/hsa-miR-3614-5p/TDGF1 ceRNA axis as a potential regulatory circuit driving colon cancer pathogenesis.

Mechanistically, TDGF1 exerts its oncogenic effects by orchestrating complex, dual intracellular signaling networks. TDGF1 undergoes fucosylation at a conserved threonine (Thr88) in its EGF-like domain, which is essential for its co-receptor function in Nodal signaling [40]. Our study demonstrates that mutating Thr88 in human TDGF1 ablates its function in the Nodal-dependent Smad2 cascade while leaving its Nodal-independent signaling intact. Importantly, we experimentally verified that Glypican-1 is strictly required for both wild-type and T88A mutant TDGF1 to fully activate downstream MAPK and Akt pathways. This functional dependence supports previous structural perspectives that the EGF-like domain mediates Glypican-1 interactions [52]. Thus, specific residues within TDGF1 determine its partner-binding specificity, governing the selective co-activation of distinct Nodal-dependent (Smad2) and Nodal-independent (Glypican-1/MAPK/AKT) signaling axes to promote malignant transformation, thereby expanding upon previous models of TDGF1 signaling [38,39,52].

Beyond cell-autonomous signaling, given that the tumor immune microenvironment critically influences clinical outcomes and therapeutic efficacy [53,54,55,56], we further elucidated the clinical and immune relevance of TDGF1. Our bioinformatic profiling indicated that elevated TDGF1 expression correlates with an immunosuppressive state, characterized by reduced infiltration of CD8^+^ T cells, neutrophils, and dendritic cells, alongside the downregulation of key immune checkpoints (such as PDCD1, CD274, CTLA4), which also aligns with prior studies suggesting TDGF1 modulates macrophage activity [57]. At the single-cell level, a powerful tool for elucidating tumor microenvironments [58], we identified a significant enrichment of the LGALS9-CD44 axis in CRC cell-T cell interactions, with CD44 strongly co-expressed with TDGF1, a relationship also observed in glioblastoma [59]. Clinically, our tissue microarray cohort validated that elevated TDGF1 serves as a negative prognostic indicator associated with advanced T stage. Intriguingly, our clinical analyses revealed that high TDGF1 levels inversely correlated with the expression of core mismatch repair (MMR) proteins (MLH1 and PMS2), which are well-documented drivers of microsatellite instability when deficient [60]. However, our in vitro evidence demonstrated that TDGF1 knockdown does not significantly alter the protein levels of MLH1 or PMS2 (Figure S17), indicating that TDGF1 does not directly regulate their expression. Therefore, we suggest that this inverse relationship represents a clinical correlation rather than a direct causal biological mechanism. Together, these findings highlight TDGF1 as a multifaceted biomarker linked to advanced tumor progression and distinct microenvironmental landscapes, rather than a direct functional driver of MMR deficiency.

Nevertheless, this study has certain limitations. First, while the in vivo oncogenic role of TDGF1 was robustly validated, the upstream ceRNA components (OLMALINC and miR-3614-5p) were primarily characterized in vitro. Fortunately, a recent study has demonstrated that exosome-mediated delivery of miR-3614-5p effectively inhibits colorectal cancer growth in vivo using xenograft models [61]. Second, our conclusions regarding the immunosuppressive microenvironment and cell–cell crosstalk, as well as the broad prognostic evaluations, rely mainly on retrospective public databases, which introduces potential analytical biases. Although our computational immune findings are partly consistent with the established immunomodulatory roles of TDGF1 [57], direct functional validations using in vitro immune co-culture systems are currently lacking. Therefore, future prospective clinical cohorts, integrated in vivo animal models evaluating the complete ceRNA axis, and deeper in vitro and in vivo immunological validations are urgently required in future studies.

## 5. Conclusions

In summary, we identify TDGF1 as a critical oncogenic driver and a prognostic biomarker in colon cancer, functioning through the OLMALINC/miR-3614-5p/TDGF1 ceRNA axis. Experimentally, TDGF1 promotes malignancy via the Nodal/Smad2 and MAPK/AKT signaling pathways. Transcriptomic analyses further reveal that elevated TDGF1 expression correlates with an immunosuppressive tumor microenvironment. Clinically, high TDGF1 levels are strongly associated with poor disease-free and overall survival. Together, these findings uncover a therapeutically actionable regulatory circuit with potential to improve patient outcomes.

## Figures and Tables

**Table 1:** Correlation analysis between TDGF1 and biomarkers of immune cells in COAD determined by GEPIA database.

Immune Cell	Biomarker	R Value	Adjusted *p* Value
B cell	CD19	−0.14 ^a^	2.72 × 10^−2^ *
	CD79A	−0.15 ^a^	1.97 × 10^−2^ *
CD8^+^ T cell	CD8A	−0.28 ^a^	2.52 × 10^−5^ **
	CD8B	−0.12	6.07 × 10^−2^
CD4^+^ T cell	CD4	−0.22 ^a^	6.30 × 10^−4^ **
M1 macrophage	NOS2	−0.17 ^a^	5.46 × 10^−3^ **
	IRF5	−0.017	7.80 × 10^−1^
	PTGS2	−0.19 ^a^	3.00 × 10^−3^ **
M2 macrophage	CD163	−0.2 ^a^	1.41 × 10^−3^ **
	VSIG4	−0.22 ^a^	7.47 × 10^−4^ **
	MS4A4A	−0.24 ^a^	2.60 × 10^−4^ **
Neutrophil	CEACAM8	−0.034	6.09 × 10^−1^
	ITGAM	−0.19 ^a^	2.91 × 10^−3^ **
	CCR7	−0.2 ^a^	1.75 × 10^−3^ **
Dendritic cell	HLA-DPB1	−0.26 ^a^	5.09 × 10^−5^ **
	HLA-DQB1	−0.22 ^a^	7.47 × 10^−4^ **
	HLA-DRA	−0.32 ^a^	1.87 × 10^−6^ **
	HLA-DPA1	−0.28 ^a^	2.52 × 10^−5^ **
	CD1C	−0.1	1.07 × 10^−1^
	NRP1	−0.2 ^a^	1.75 × 10^−3^ **
	ITGAX	−0.22 ^a^	6.30 × 10^−4^ **

^a^ These results are statistically significant. * *p* value < 0.05; ** *p* value < 0.01.

## Data Availability

The data presented in this study are openly available in the TCGA (The Cancer Genome Atlas) repository at https://portal.gdc.cancer.gov/ (accessed on 16 April 2022), with project ID TCGA-COAD and dbGaP Study Accession phs000178. All other data supporting the findings are available within the paper and/or its Supplementary Information.

## Supplementary Materials

The following supporting information can be downloaded at: https://www.mdpi.com/article/10.3390/cells15131141/s1, Figure S1: Pan-cancer expression profiling of TDGF1; Figure S2: Identification of TDGF1-targeting miRNAs in colon cancer; Figure S3: Characterization of upstream lncRNAs regulating hsa-miR-3614-5p in colon cancer; Figure S4: Subcellular localization of OLMALINC in colon cancer cells; Figure S5: Validation of transfection efficiency for the functional rescue experiments; Figure S6: The relationship between TDGF1 level and immune cell infiltration in colon cancer; Figure S7: TDGF1 co-expression networks and functional enrichment in colon cancer analyzed by the LinkedOmics database; Figure S8: Expression profiles of TDGF1 and cellular composition in CRC single-cell samples; Figure S9: Whole expression profiles of TDGF1 in additional CRC single-cell samples; Figure S10: TDGF1 expression levels across cell subtypes in additional CRC single-cell samples; Figure S11: Functional annotation of TDGF1 co-expression networks in CRC single-cell data; Figure S12: Protein-protein interaction networks of DEGs in CRC single-cell samples stratified by TDGF1 expression; Figure S13: Cell-cell interaction networks for CRC single-cell samples (Cohort 1); Figure S14: Cell-cell interaction networks for CRC single-cell samples (Cohort 2); Figure S15: Ligand-receptor analysis in malignant cell communication networks (Cohort 1); Figure S16: Ligand-receptor analysis in malignant cell communication networks (Cohort 2); Figure S17: TDGF1 knockdown does not directly alter the protein expression of mismatch repair proteins MLH1 and PMS2 in vitro; Table S1: Primer sequences for qRT-PCR detection; Table S2: Correlation analysis between TDGF1 and miRNAs in COAD determined by ENCORI database; Table S3: Correlation analysis between hsa-miR-3614-5p and lncRNAs determined by ENCORI database; Table S4: Correlation analysis between TDGF1 and lncRNAs determined by ENCORI database; Table S5: TDGF1 co-expression genes in colon cancer analyzed by LinkedOmics database; Table S6: TDGF1 co-expression genes in CRC single-cell samples analyzed by CancerSCEM database; Table S7: Top 20 clusters in pathway and process enrichment analysis of TDGF1 associated DEGs in CRC single-cell samples analyzed by Metascape database.

## Abbreviations

The following abbreviations are used in this manuscript:

TDGF1	Teratocarcinoma-derived growth factor 1
ceRNA	Competing endogenous RNA
COAD	Colon adenocarcinoma
EMT	Epithelial to mesenchymal transition
OS	Overall survival
DFS	Disease-free survival
ncRNAs	Noncoding RNAs
miRNAs	microRNAs
lncRNAs	Long noncoding RNAs
TIME	Tumor immune microenvironment
scRNA-seq	Single-cell RNA sequencing
TCGA	The Cancer Genome Atlas
TPM	Transcripts per million
ROC	Receiver operating characteristic
AUC	Area under the curve
CPTAC	Clinical Proteomic Tumor Analysis Consortium
GO_BP	Gene Ontology biological process
GSEA	Gene set enrichment analysis
DEGs	Differentially expressed genes
PPI	Protein–protein interaction
wt	Wild-type
mut	Mutant
qRT-PCR	Quantitative real-time PCR
ECL	Enhanced chemiluminescence
IHC	Immunohistochemistry
SD	Standard deviation
HRs	Hazard ratios
CIs	Confidence intervals
sCNAs	Somatic copy number alterations
circRNAs	Circular RNAs
