# Supplementary material for: TDGF1 Mediates the Oncogenic Effects of the OLMALINC/miR-3614-5p ceRNA Axis in Colon Cancer Through Nodal/Smad2 and Glypican-1/MAPK-AKT Signaling
Source: Cells. 2026 Jun 23;15(13):1141. doi: 10.3390/cells15131141 (PMC13360589; doi:10.3390/cells15131141)
Supplement: Supplementary file 1 [file cells-15-01141-s001.zip › cells-4350048-supplementary.pdf]

Supplementary Materials for

TDGF1 mediates the oncogenic effects of the OLMALINC/miR-3614-5p ceRNA axis in colon cancer through Nodal/Smad2 and Glypican-1/MAPK-AKT signaling

*Corresponding author: Xiaoguang Yang, yangxg168@nenu.edu.cn

This PDF file includes:

Fig. S1 to S17

Table S1 toS7

Supplementary Figures

Figure S1

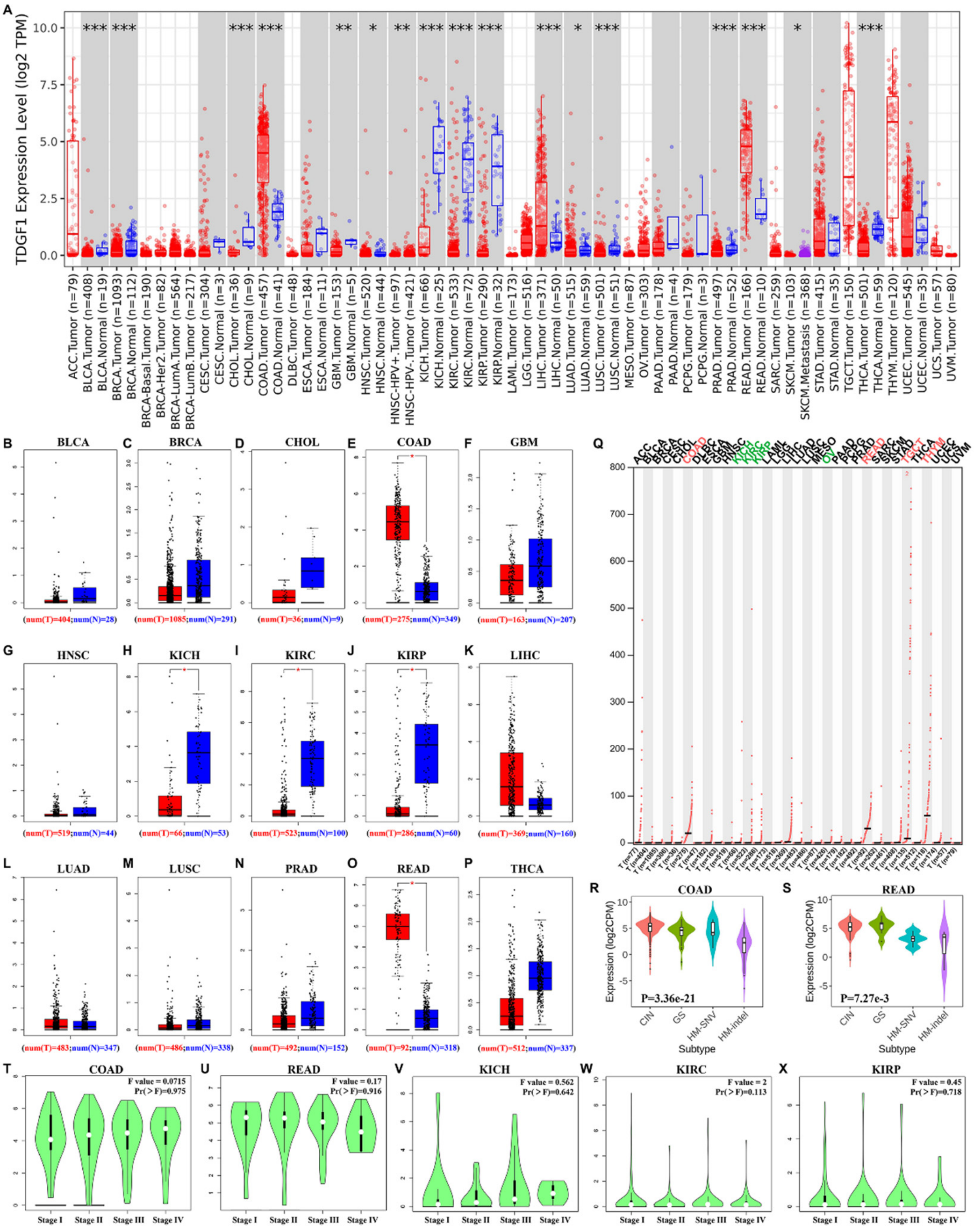

Figure S1. Pan-cancer expression profiling of TDGF1.

(A) TDGF1 mRNA expression in tumor tissues versus matched adjacent non-tumor tissues across TCGA cancers. Data presented as mean ± SD. Significance was calculated with Student's t test. *p < 0.05, **p < 0.01, ***p < 0.001.

(B–P) TDGF1 expression in tumor tissues (TCGA) versus combined normal controls (TCGA +

GTEx) for BLCA, BRCA, CHOL, COAD, GBM, HNSC, KICH, KIRC, KIRP, LIHC, LUAD, LUSC, PRAD, READ, and THCA. Data presented as mean ± SD. Significance was calculated with Student's t test. Bars without symbols indicate no significant difference, *$p < 0.05$.

(Q) Pan-cancer expression landscape of TDGF1 based solely on TCGA data.

(R-S) TDGF1 mRNA levels across molecular or histological subtypes of COAD (R) and READ (S). Data presented as mean ± SD. Significance was calculated with one-way ANOVA.

(T-X) TDGF1 expression across different pathological stages of COAD, READ, KICH, KIRC and KIRP. Data presented as mean ± SD. Significance was calculated with one-way ANOVA.

Figure S2

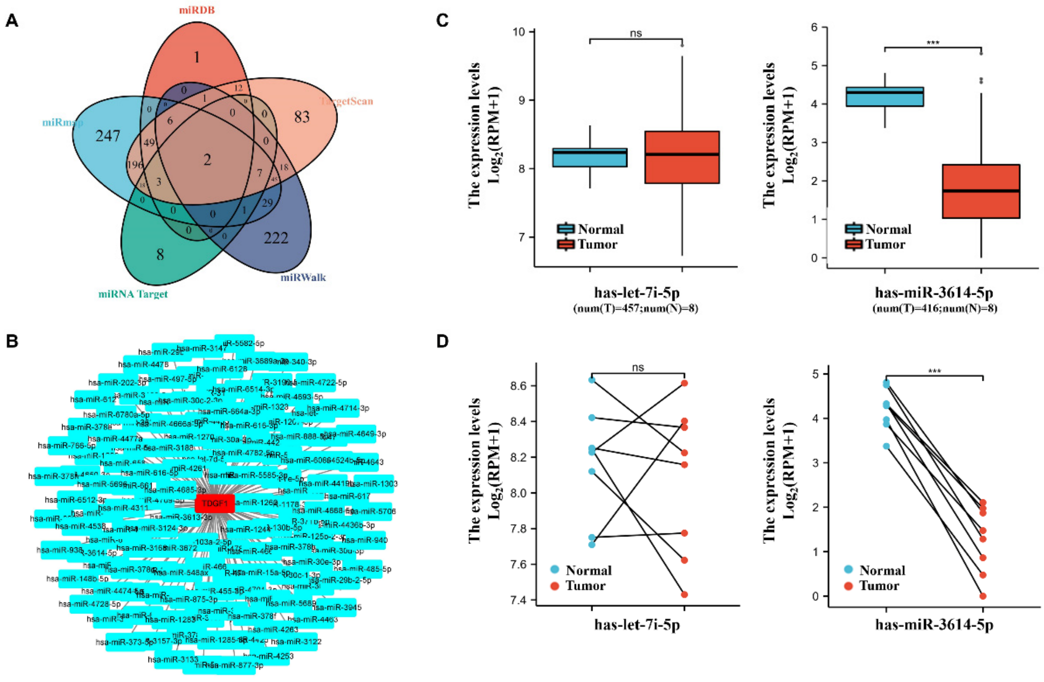

Figure S2. Identification of TDGF1-targeting miRNAs in colon cancer.

(A) Venn diagram showing the overlap of predicted miRNAs targeting TDGF1 from five databases: TargetScan, miRDB, miRmap, miR Target, and miRWalk.

(B) Regulatory network of miRNA-TDGF1 interactions visualized using Cytoscape software. Nodes represent miRNAs (blue) and TDGF1 (red); edges indicate predicted targeting relationships.

(C-D) Expression levels of candidate miRNAs in (C) unpaired COAD tumors vs. normal tissues and (D) paired tumor-adjacent normal specimens from the TCGA dataset. Data presented as mean ± SD. Significance was calculated with Student's t test. ***p < 0.001, ns, no significance.

Figure S3

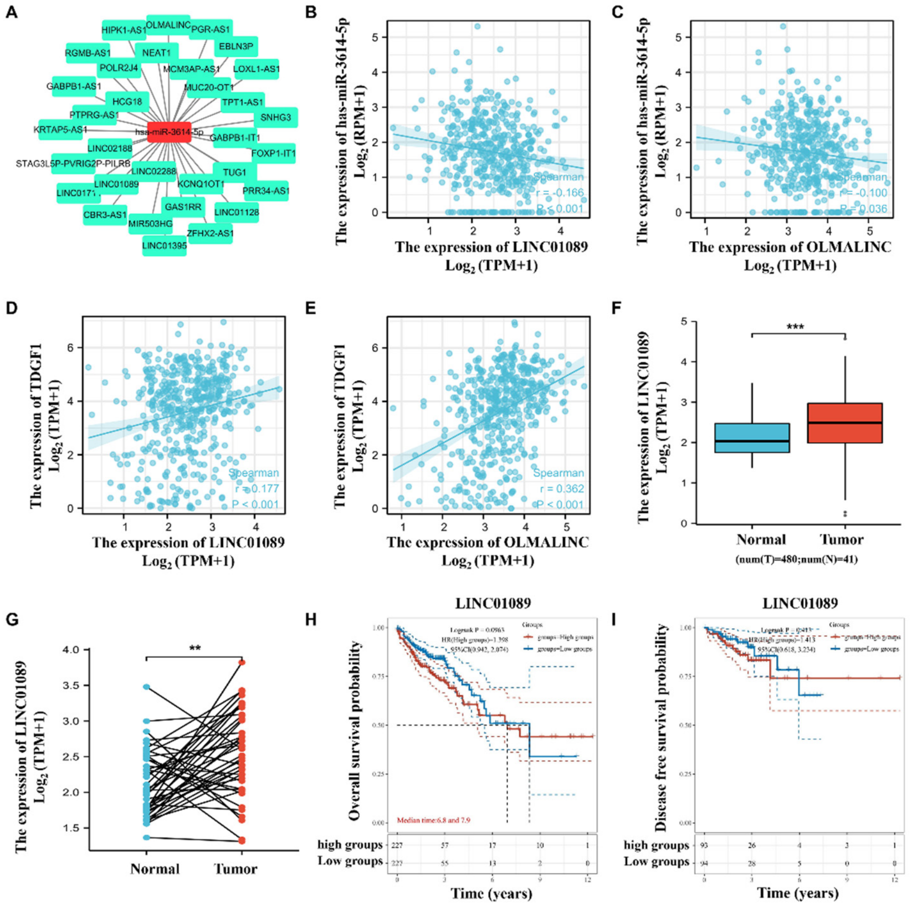

Figure S3. Characterization of upstream lncRNAs regulating hsa-miR-3614-5p in colon cancer.

(A) The lncRNA-hsa-miR-3614-5p regulatory network visualized using Cytoscape software.

(B-E) Spearman correlation analysis between (B, C) LINC01089 or (D, E) OLMALINC and hsa-miR-3614-5p expression in COAD samples from the ENCORI database. Correlation coefficient (R) and p-value were shown.

(F-G) Expression levels of LINC01089 in unpaired (F, G) and paired (H, I) COAD tumor/normal specimens from the TCGA dataset. Data presented as mean ± SD. Significance was calculated with Student's t test. $^{**}p < 0.01$, $^{***}p < 0.001$.

(H-I) Kaplan-Meier curves for OS and DFS of COAD patients stratified by high (red) and low (blue) expression of LINC01089. Log-rank p-values were indicated.

Figure S4

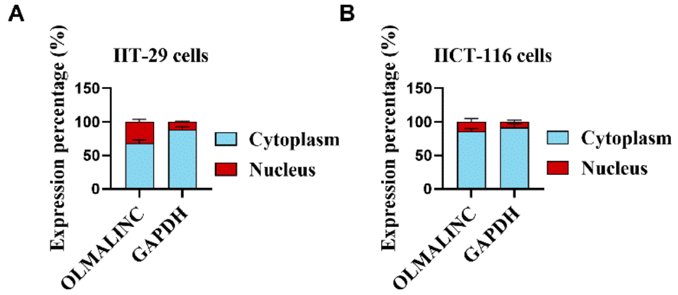

Figure S4. Subcellular localization of OLMALINC in colon cancer cells.

(A-B) Cellular fractionation coupled with qRT-PCR analysis was performed to determine the relative distribution of OLMALINC in the cytoplasmic (light blue) and nuclear (red) fractions of HT-29 (A) and HCT-116 (B) cells. GAPDH was evaluated simultaneously as a standard cytoplasmic control. The bar graphs represent the percentage of the indicated RNAs in each subcellular compartment. Data are presented as mean ± SD of three independent experiments (n = 3).

Figure S5

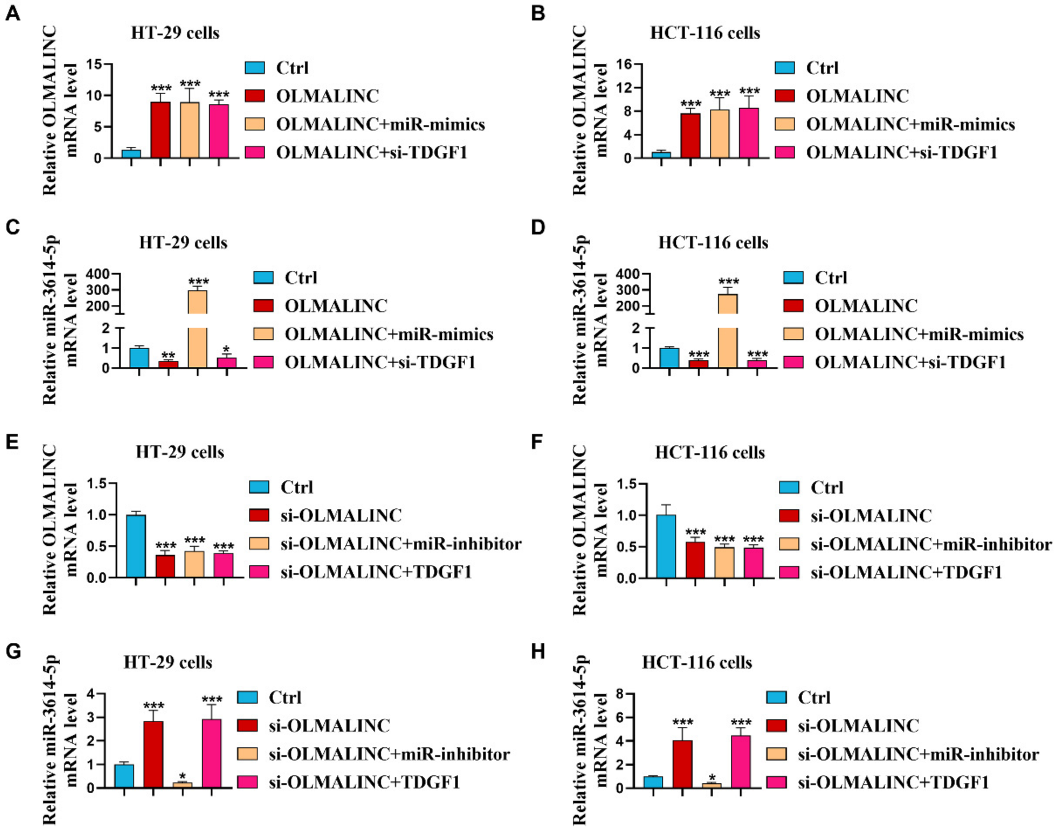

Figure S5. Validation of transfection efficiency for the functional rescue experiments.

(A-D) The relative expression levels of OLMALINC (A-B) and hsa-miR-3614-5p (C-D) were measured by qRT-PCR in HT-29 and HCT-116 cells co-transfected with OLMALINC overexpression plasmid along with either hsa-miR-3614-5p mimics or si-TDGF1.

(E-H) The relative expression levels of OLMALINC (E-F) and hsa-miR-3614-5p (G-H) were determined by qRT-PCR in HT-29 and HCT-116 cells co-transfected with si-OLMALINC along with either hsa-miR-3614-5p inhibitor or TDGF1 overexpression plasmid.

All data were normalized to internal controls and presented as the mean ± SD from three independent experiments (n = 3). *$p < 0.05$, **$p < 0.01$, and ***$p < 0.001$ vs. the Ctrl group.

Figure S6

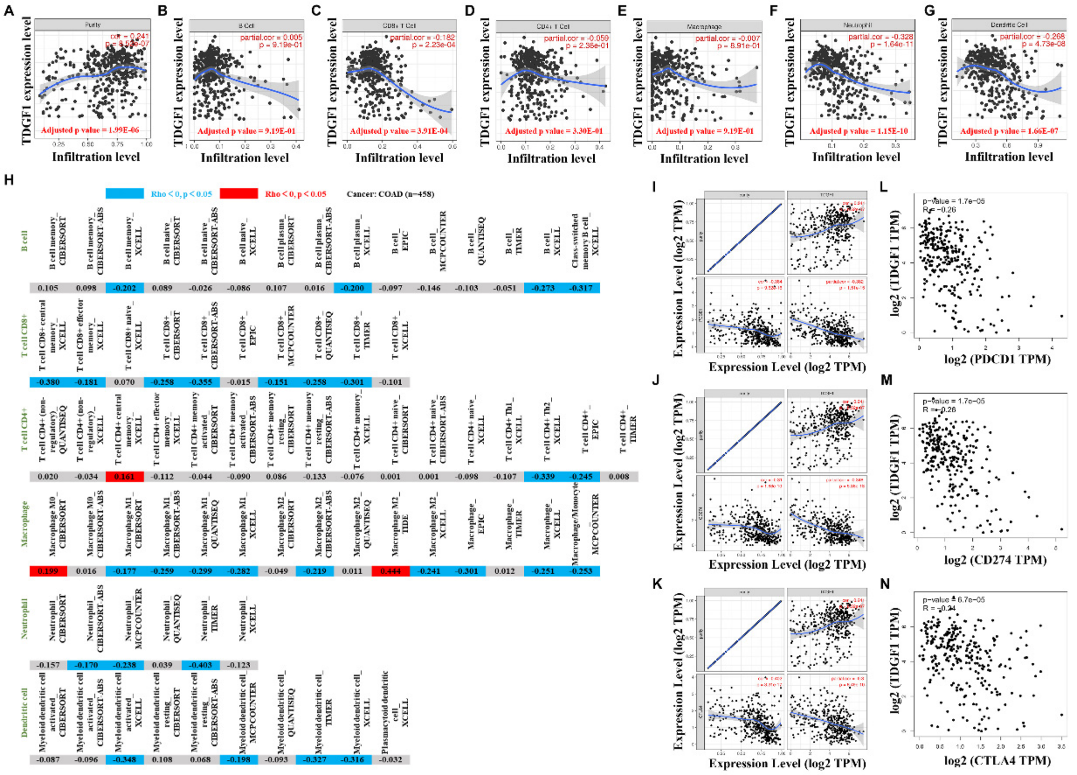

Figure S6. The relationship between TDGF1 level and immune cell infiltration in colon cancer.

(A) Correlation analysis between TDGF1 expression and tumor purity in COAD via TIMER database.

(B–G) Correlation analyses between TDGF1 expression and infiltration levels of B cells (B), CD8+ T cells (C), CD4+ T cells (D), macrophages (E), neutrophils (F), and dendritic cells (G) in COAD via TIMER database. Partial.cor and p-values were shown.

(H) Subtype-specific analysis of immune infiltration signatures correlated with TDGF1 expression via TIMER database. Spearman's correlation coefficient (Rho) and p-values were shown.

(I-K) Spearman correlation between TDGF1 and PDCD1, CD274, or CTLA4 in COAD, adjusted for tumor purity using TIMER database. Partial.cor and p-values were shown.

(L-N) Pearson correlation between TDGF1 expression and PDCD1, CD274 or CTLA4 in COAD from the GEPIA database. Correlation coefficient (R) and p-values were shown.

Figure S7

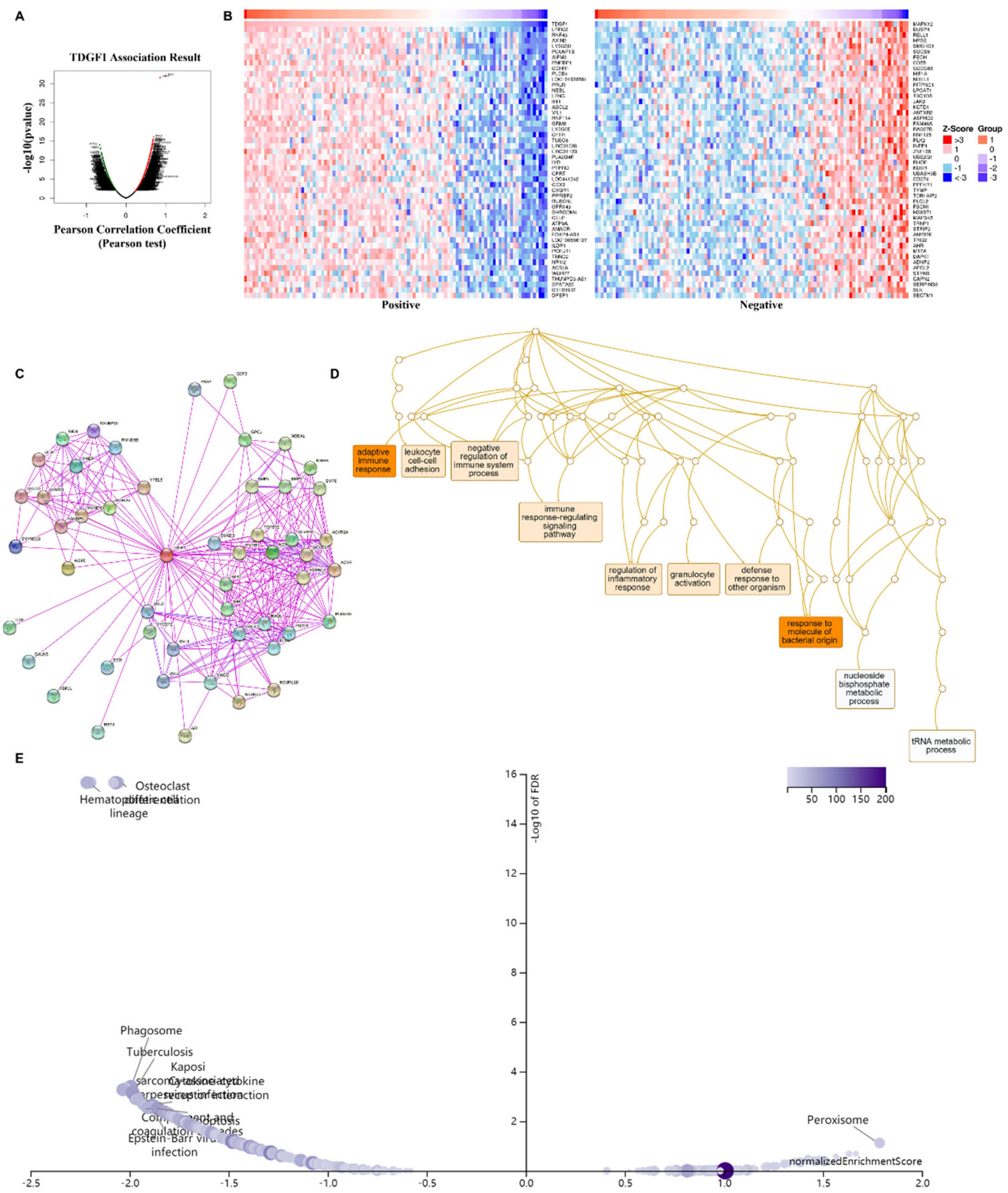

Figure S7. TDGF1 co-expression networks and functional enrichment in colon cancer analyzed by the LinkedOmics database.

(A) Volcano plot showing genes positively (red) and negatively (green) correlated with TDGF1 expression in COAD (Pearson correlation, FDR < 0.01).

(B) Heatmap of the top 50 genes positively and negatively correlated with TDGF1 expression in COAD.

(C) Protein-protein interaction (PPI) network of experimentally validated TDGF1 interactors from

the STRING database.

(D) Gene Ontology (GO) Biological Process enrichment analysis for TDGF1-correlated genes.

(E) KEGG pathway enrichment analysis (Volcano plot) for TDGF1-associated genes.

Figure S8

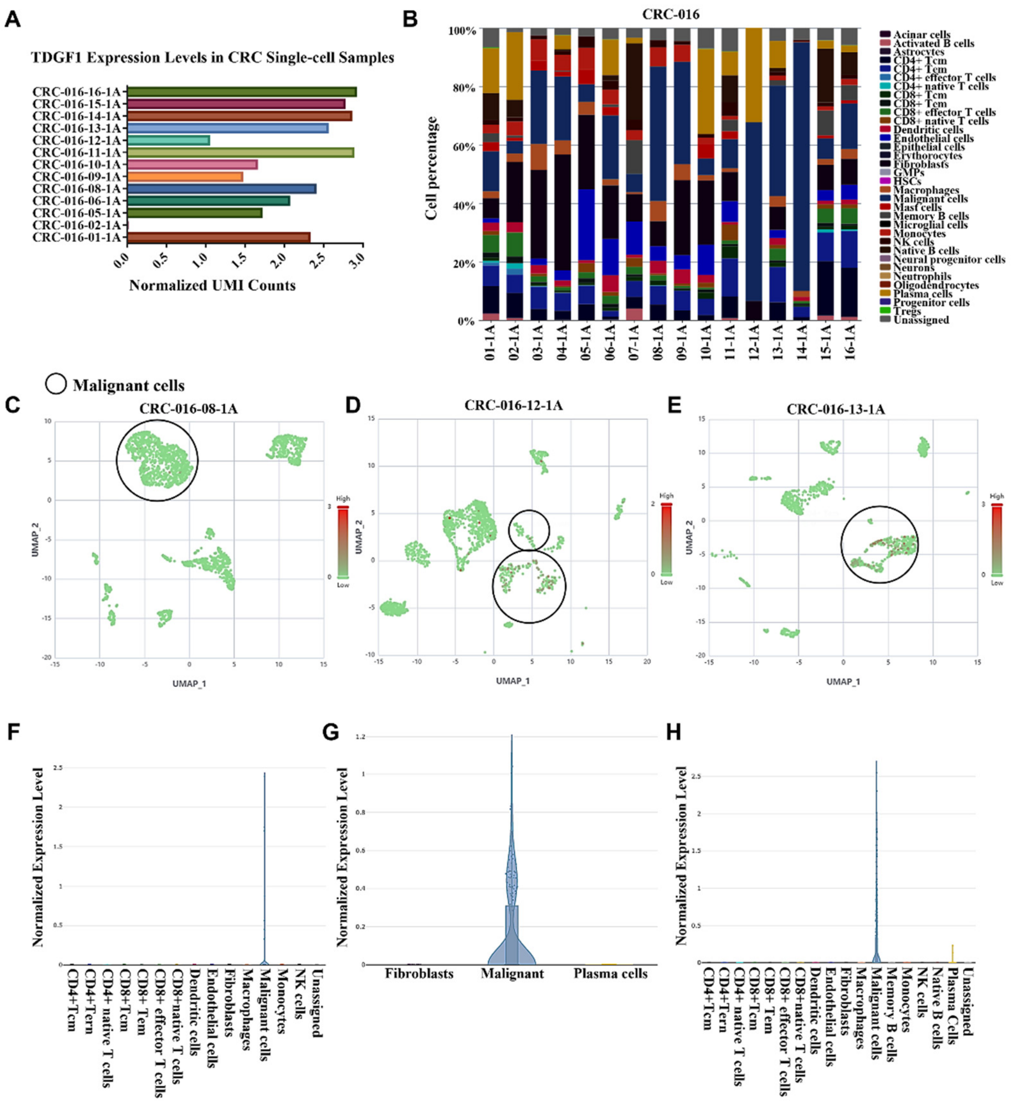

Figure S8. Expression profiles of TDGF1 and cellular composition in CRC single-cell samples.

(A) Histograms showing TDGF1 expression levels across major cell types in the integrated CRC single-cell cohort from the CancerSCEM database.

(B) Stacked bar chart comparing the relative proportions of major cell types among different CRC single-cell samples.

(C-E) Uniform Manifold Approximation and Projection (UMAP) plots visualizing the whole expression landscape of TDGF1 (color gradient) in samples CRC-016-08-1A (C), CRC-016-12-1A (D), and CRC-016-13-1A (E).

(F-H) Violin plots showing TDGF1 expression levels across annotated cell subtypes in samples CRC-016-08-1A (F), CRC-016-12-1A (G), and CRC-016-13-1A (H).

Figure S9

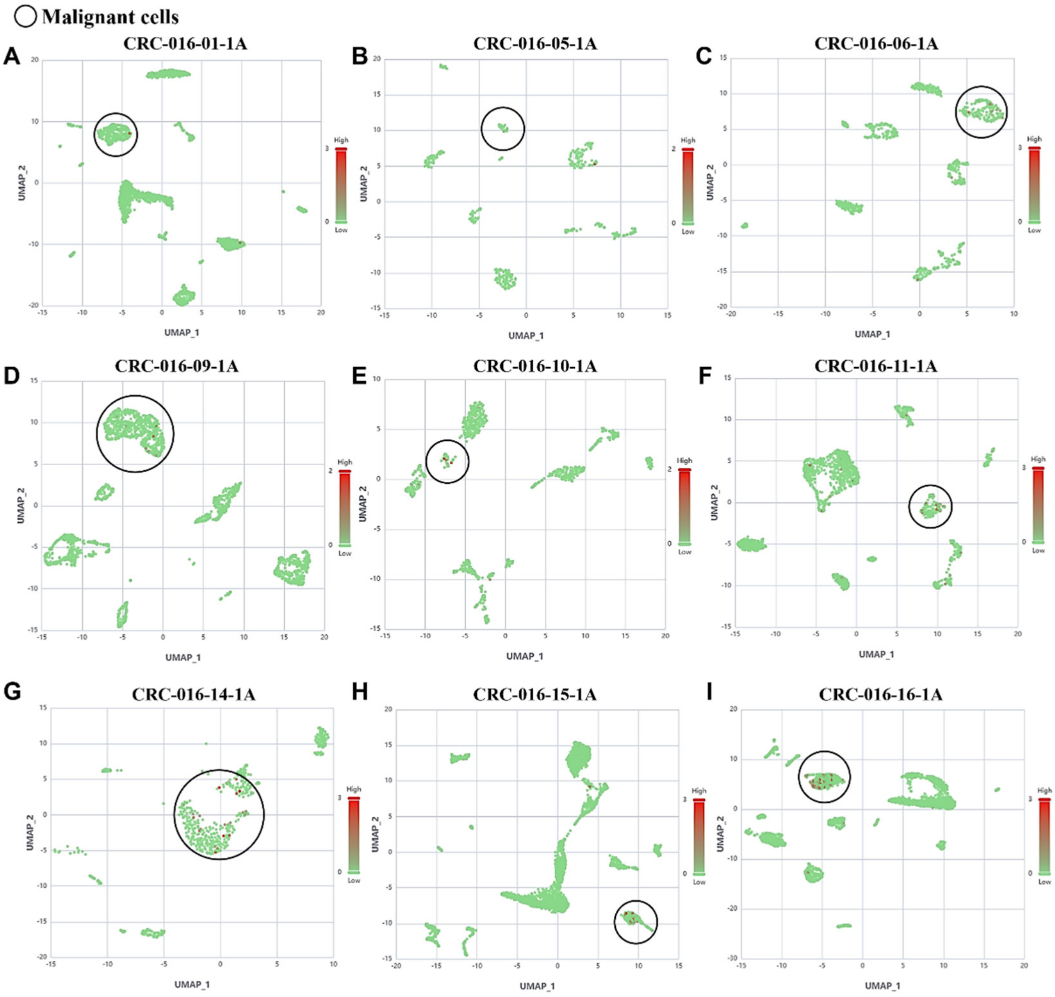

Figure S9. Whole expression profiles of TDGF1 in additional CRC single-cell samples.

(A-I) UMAP plots visualizing the expression landscape of TDGF1 (color gradient) in CRC single-cell samples: CRC-016-01-1A (A), CRC-016-05-1A (B), CRC-016-06-1A (C), CRC-016-09-1A (D), CRC-016-10-1A (E), CRC-016-11-1A (F), CRC-016-14-1A (G), CRC-016-15-1A (H), and CRC-016-16-1A (I). Data sourced from the CancerSCEM database.

Figure S10

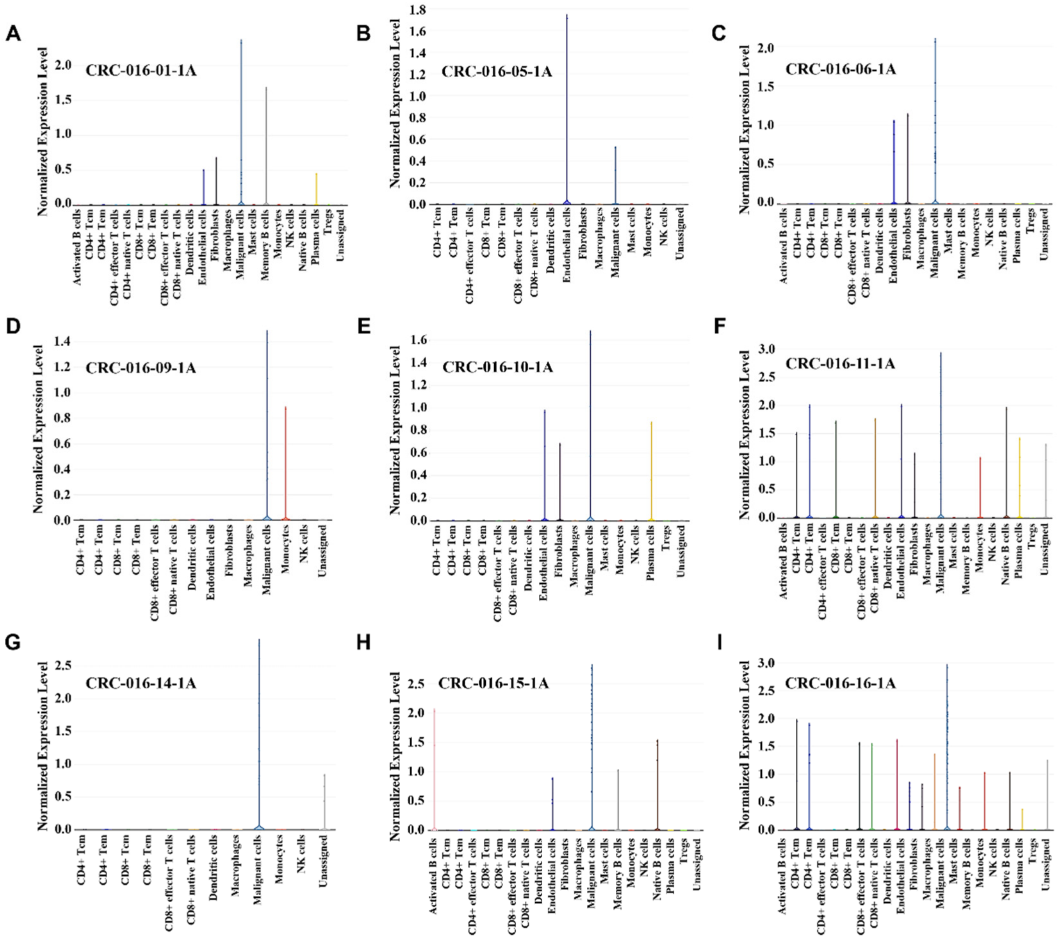

Figure S10. TDGF1 expression levels across cell subtypes in additional CRC single-cell samples.

(A-I) Violin plots showing the distribution of TDGF1 expression levels within annotated cell subtypes for samples: CRC-016-01-1A (A), CRC-016-05-1A (B), CRC-016-06-1A (C), CRC-016-09-1A (D), CRC-016-10-1A (E), CRC-016-11-1A (F), CRC-016-14-1A (G), CRC-016-15-1A (H), and CRC-016-16-1A (I).

Figure S11

A

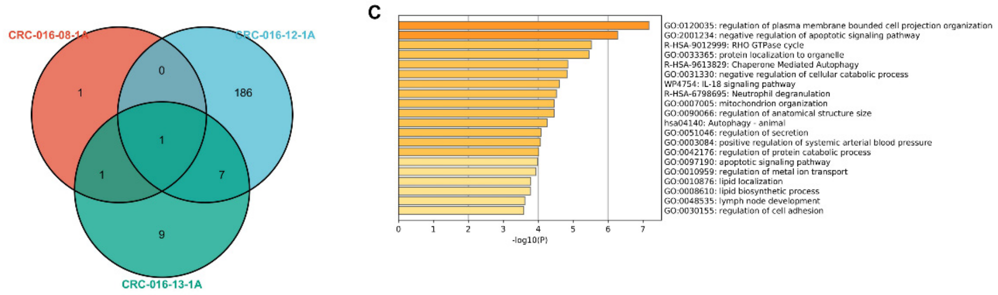

B

Target Gene	Related Gene	Correlation Coefficient (Pearson)	P-value	Sample ID
TDGF1	NDUFAB1	0.044	0.042	CRC-016-08-1A
		0.13	0.026	CRC-016-12-1A
		0.148	0.000	CRC-016-13-1A
	TSPAN6	0.329	0.000	CRC-016-12-1A
		0.116	0.000	CRC-016-13-1A
	DPM1	0.249	0.000	CRC-016-12-1A
		0.075	0.026	CRC-016-13-1A
	M6PR	0.22	0.000	CRC-016-12-1A
		-0.064	0.046	CRC-016-13-1A
	FKBP4	0.207	0.000	CRC-016-12-1A
		0.15	0.000	CRC-016-13-1A
	CFTR	0.18	0.002	CRC-016-12-1A
		0.064	0.046	CRC-016-13-1A
	ICA1	0.152	0.010	CRC-016-12-1A
		0.146	0.000	CRC-016-13-1A
	CD38	-0.175	0.004	CRC-016-12-1A
		-0.081	0.018	CRC-016-13-1A
	AOC1	0.045	0.038	CRC-016-08-1A
		0.09	0.009	CRC-016-13-1A

D

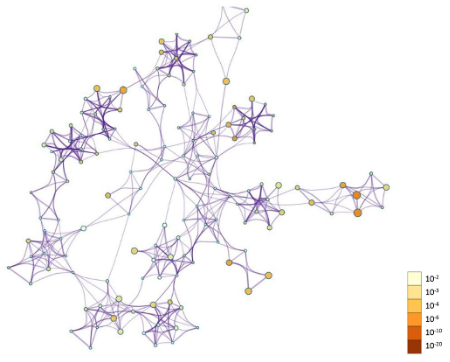

Figure S11. Functional annotation of TDGF1 co-expression networks in CRC single-cell data.

(A) Venn diagram showing shared and distinct differentially expressed genes (DEGs) associated with TDGF1 across single-cell samples from the CancerSCEM database.

(B) Correlation between predicted DEGs and TDGF1 expression in CRC single-cell samples.

(C) The statistically enriched terms were identified and then hierarchically clustered into a tree based on the threshold of kappa score as 0.3.

(D) Enrichment network with nodes colored by the statistical significance (-log10(p-value)).

Figure S12

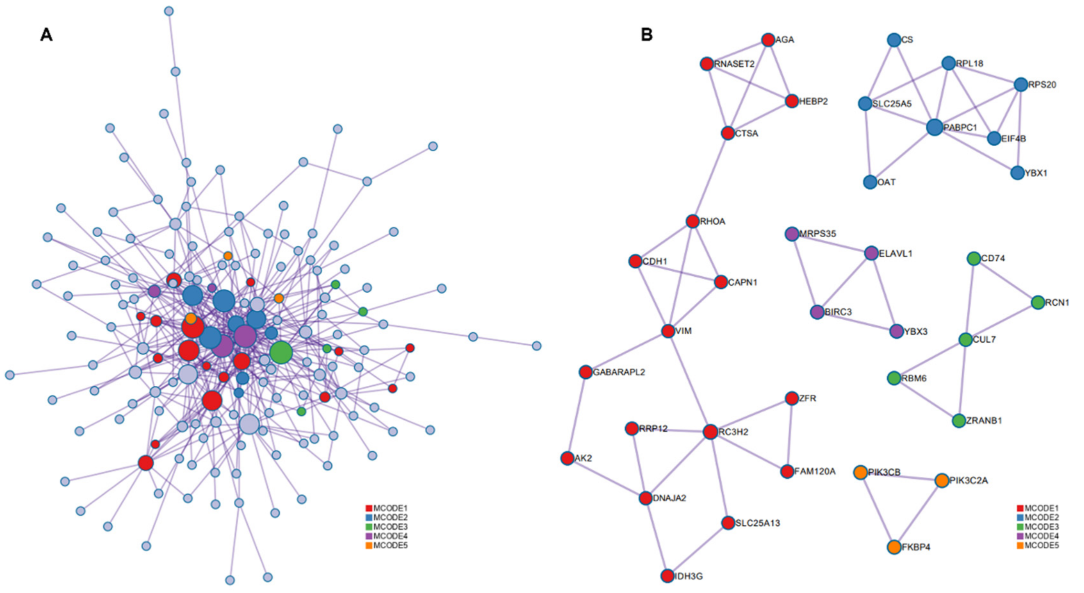

Figure S12. Protein-protein interaction networks of DEGs in CRC single-cell samples stratified by TDGF1 expression.

(A-B) Molecular Complex Detection (MCODE) networks generated from the protein-protein interaction (PPI) analysis of DEGs identified in single cells with high or low TDGF1 expression in CancerSCEM database.

Figure S13

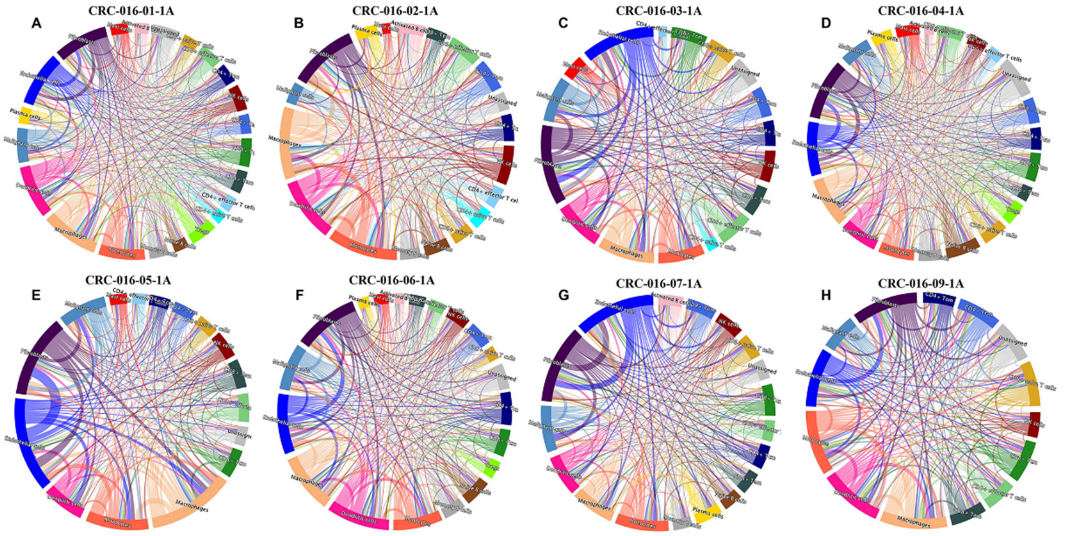

Figure S13. Cell-cell interaction networks for CRC single-cell samples (Cohort 1).

(A-H) Circle plots depicting cell-cell communication networks for samples CRC-016-01-1A (A), CRC-016-02-1A (B), CRC-016-03-1A (C), CRC-016-04-1A (D), CRC-016-05-1A (E), CRC-016-06-1A (F), CRC-016-07-1A (G), and CRC-016-09-1A (H) in CancerSCEM database.

Figure S14

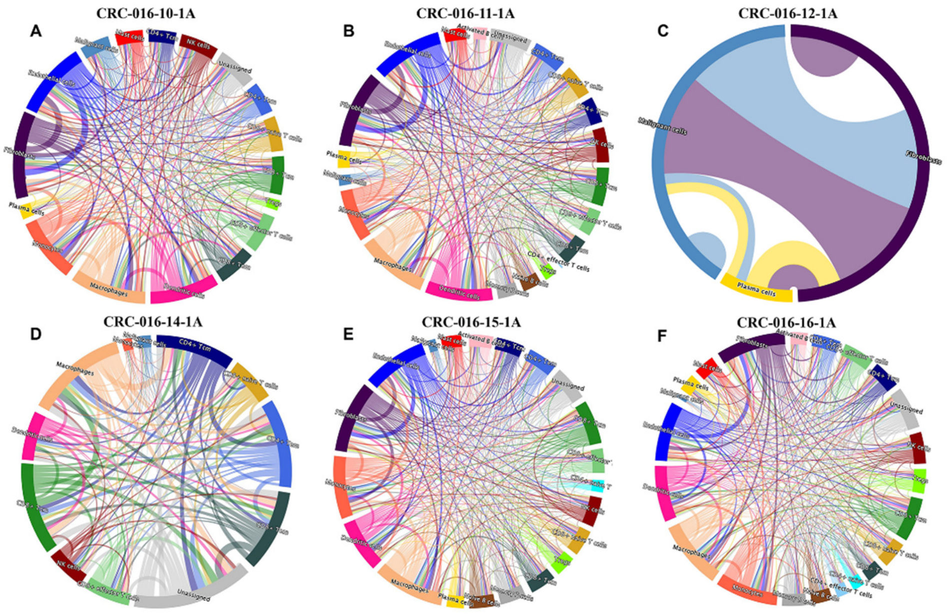

Figure S14. Cell-cell interaction networks for CRC single-cell samples (Cohort 2).

(A-F) Circle plots depicting cell-cell communication networks for samples CRC-016-10-1A (A), CRC-016-11-1A (B), CRC-016-12-1A (C), CRC-016-14-1A (D), CRC-016-15-1A (E), and CRC-016-16-1A (F) in CancerSCEM database.

Figure S15

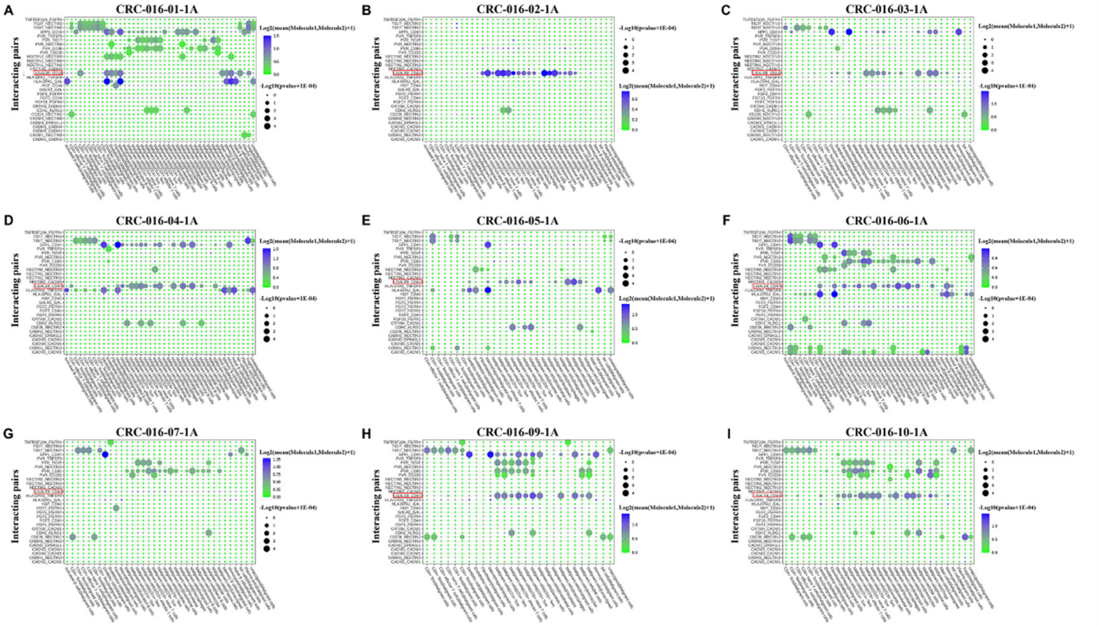

Figure S15. Ligand-receptor analysis in malignant cell communication networks (Cohort 1). **(A-I)** Dot plots summarizing significant ligand-receptor pairs mediating interactions between malignant cells and other cell types in samples CRC-016-01-1A (A), CRC-016-02-1A (B), CRC-016-03-1A (C), CRC-016-04-1A (D), CRC-016-05-1A (E), CRC-016-06-1A (F), CRC-016-07-1A (G), CRC-016-09-1A (H), and CRC-016-10-1A (I) in CancerSCEM database. Dot size represents the communication probability, and color indicates the computed p-value.

Figure S16

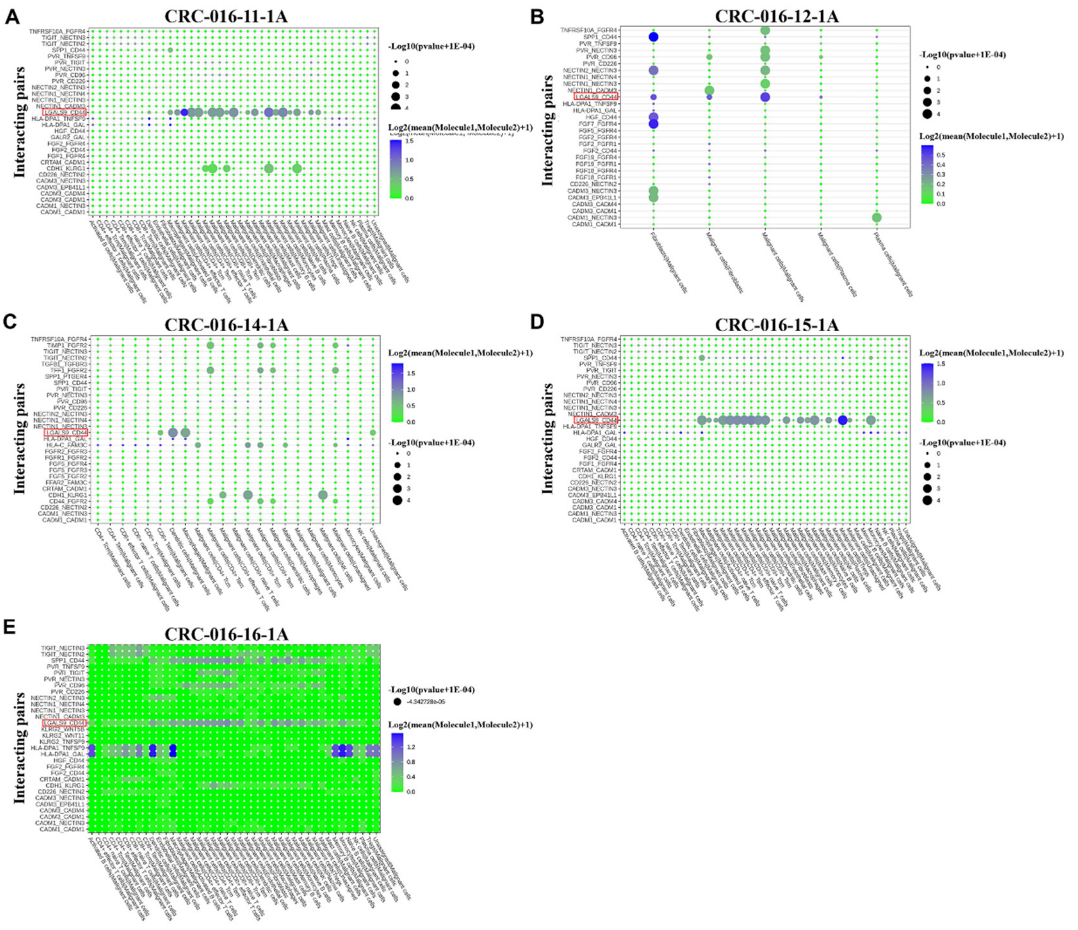

Figure S16. Ligand-receptor analysis in malignant cell communication networks (Cohort 2). (A-E) Dot plots summarizing significant ligand-receptor pairs for interactions involving malignant cells in samples CRC-016-11-1A (A), CRC-016-12-1A (B), CRC-016-14-1A (C), CRC-016-15-1A (D), and CRC-016-16-1A (E) in CancerSCEM database. Dot size represents the communication probability, and color indicates the computed p-value.

Figure S17

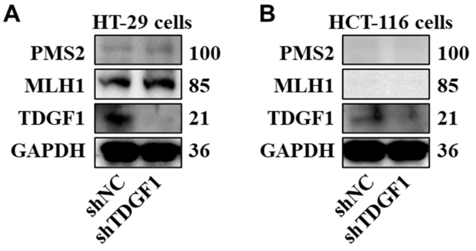

Figure S17. TDGF1 knockdown does not directly alter the protein expression of mismatch repair proteins MLH1 and PMS2 *in vitro*.

(A-B) Western blot analysis evaluating the protein levels of MMR core components, PMS2 and MLH1, following TDGF1 knockdown. HT-29 (A) and HCT-116 (B) colon cancer cells were transfected with either TDGF1-specific short hairpin RNA (shTDGF1) or a negative control (shNC). GAPDH served as loading control. N = 3 biological replication.

Supplementary Tables

Table S1. Primer sequences for qRT-PCR detection

Symbol	Primer	Primer Sequence (5′–3′)
has-miR-3614-5p	F-Primer	ACACTCCAGCTGGGCCACTTGGATCTGAAGG
	R-Primer	TGGTGTCGTGGAGTCG
OLMALINC	F-Primer	AGTCTGCCCTACCCTGGAAT
	R-Primer	CCCGTCTGGAAAACTCTCTG
U6	F-Primer	CTCGCTTCGGCAGCACA
	R-Primer	AACGCTTCACGAATTTGCGT
GAPDH	F-Primer	ACAACTTTGGTATCGTGGAAGG
	R-Primer	GCCATCACGCCACAGTTTC

Table S2. Correlation analysis between TDGF1 and miRNAs in COAD determined by ENCORI database

Gene	miRNA	R value	Adjusted p value
TDGF1	hsa-miR-4478	-0.015	1.000
TDGF1	hsa-miR-4728-5p	-0.046	0.944
TDGF1	hsa-miR-485-5p	0.044	0.954
TDGF1	hsa-miR-3929	-0.051	0.909
TDGF1	hsa-miR-4722-5p	-0.006	1.000
TDGF1	hsa-miR-3689a-3p	0	1.000
TDGF1	hsa-miR-4643	0.062	0.822
TDGF1	hsa-miR-4701-3p	0.034	1.000
TDGF1	hsa-miR-3945	0	1.000
TDGF1	hsa-miR-3160-5p	0.022	1.000
TDGF1	hsa-miR-340-3p	-0.05	0.909
TDGF1	hsa-miR-5093	-0.01	1.000
TDGF1	hsa-miR-30a-3p	0.031	1.000
TDGF1	hsa-miR-5584-3p	0	1.000
TDGF1	hsa-miR-1178-3p	0.022	1.000
TDGF1	hsa-miR-371b-5p	-0.064	0.822
TDGF1	hsa-miR-3613-3p	0.074	0.813
TDGF1	hsa-miR-4684-5p	0.143[a]	0.045[*]
TDGF1	hsa-miR-4786-5p	0.032	1.000
TDGF1	hsa-miR-1207-5p	0.051	0.909
TDGF1	hsa-miR-4436b-3p	-0.047	0.944
TDGF1	hsa-miR-3188	0.045	0.946
TDGF1	hsa-miR-15a-5p	-0.049	0.909
TDGF1	hsa-miR-497-5p	0.061	0.822
TDGF1	hsa-miR-1285-3p	0.062	0.822
TDGF1	hsa-miR-548ax	0.047	0.944
TDGF1	hsa-miR-30c-1-3p	0.06	0.829
TDGF1	hsa-miR-378b	0.066	0.822
TDGF1	hsa-miR-4524b-5p	0	1.000
TDGF1	hsa-miR-378h	-0.018	1.000
TDGF1	hsa-miR-30c-2-3p	0.024	1.000
TDGF1	hsa-miR-378f	-0.01	1.000
TDGF1	hsa-miR-378a-3p	0.133	0.070
TDGF1	hsa-miR-378d	0.077	0.813
TDGF1	hsa-miR-378c	0.2[a]	0.001[**]
TDGF1	hsa-miR-378e	0.024	1.000
TDGF1	hsa-miR-1911-3p	-0.067	0.822
TDGF1	hsa-miR-6514-3p	0.029	1.000
TDGF1	hsa-miR-3122	0.011	1.000
TDGF1	hsa-miR-3130-5p	0.016	1.000

TDGF1	hsa-miR-4733-3p	0.018	1.000
TDGF1	hsa-miR-1270	-0.064	0.822
TDGF1	hsa-miR-6512-3p	0.025	1.000
TDGF1	hsa-miR-1303	-0.017	1.000
TDGF1	hsa-miR-617	0.035	1.000
TDGF1	hsa-miR-4425	0.025	1.000
TDGF1	hsa-miR-4463	0.035	1.000
TDGF1	hsa-let-7i-5p	-0.271[a]	0.000**
TDGF1	hsa-miR-3685	-0.029	1.000
TDGF1	hsa-miR-103a-2-5p	-0.05	0.909
TDGF1	hsa-let-7e-5p	-0.05	0.909
TDGF1	hsa-let-7d-5p	-0.096	0.528
TDGF1	hsa-miR-125b-2-3p	-0.024	1.000
TDGF1	hsa-miR-938	-0.013	1.000
TDGF1	hsa-miR-4714-3p	-0.018	1.000
TDGF1	hsa-miR-3157-3p	0.087	0.602
TDGF1	hsa-miR-877-3p	-0.034	1.000
TDGF1	hsa-miR-4685-3p	0	1.000
TDGF1	hsa-miR-6086	0	1.000
TDGF1	hsa-miR-130b-5p	-0.025	1.000
TDGF1	hsa-miR-29b-1-5p	0.031	1.000
TDGF1	hsa-miR-29b-2-5p	-0.144[a]	0.045*
TDGF1	hsa-miR-616-3p	-0.065	0.822
TDGF1	hsa-miR-6780a-5p	-0.017	1.000
TDGF1	hsa-miR-612	-0.02	1.000
TDGF1	hsa-miR-3187-5p	-0.137	0.058
TDGF1	hsa-miR-4419b	0.071	0.822
TDGF1	hsa-miR-4649-3p	-0.074	0.813
TDGF1	hsa-miR-661	-0.05	0.909
TDGF1	hsa-miR-3622b-5p	-0.01	1.000
TDGF1	hsa-miR-940	0.026	1.000
TDGF1	hsa-miR-3689c	0.005	1.000
TDGF1	hsa-miR-4253	0	1.000
TDGF1	hsa-miR-4768-3p	-0.041	1.000
TDGF1	hsa-miR-1262	0.013	1.000
TDGF1	hsa-miR-890	-0.037	1.000
TDGF1	hsa-miR-1827	0	1.000
TDGF1	hsa-miR-935	-0.095	0.528
TDGF1	hsa-miR-3672	0	1.000
TDGF1	hsa-miR-3675-3p	0	1.000
TDGF1	hsa-miR-548c-3p	0	1.000
TDGF1	hsa-miR-4317	0	1.000
TDGF1	hsa-miR-1299	0	1.000
TDGF1	hsa-miR-148b-5p	-0.038	1.000

TDGF1	hsa-miR-4423-3p	0.021	1.000
TDGF1	hsa-miR-373-5p	0.021	1.000
TDGF1	hsa-miR-616-5p	-0.055	0.909
TDGF1	hsa-miR-664a-3p	0.077	0.813
TDGF1	hsa-miR-3133	-0.014	1.000
TDGF1	hsa-miR-875-3p	0.027	1.000
TDGF1	hsa-miR-3124-3p	-0.015	1.000
TDGF1	hsa-miR-5582-5p	0.02	1.000
TDGF1	hsa-miR-4668-5p	0	1.000
TDGF1	hsa-miR-4666a-3p	0	1.000
TDGF1	hsa-miR-4666a-5p	-0.091	0.602
TDGF1	hsa-miR-338-5p	-0.157[a]	0.029[*]
TDGF1	hsa-miR-4311	0	1.000
TDGF1	hsa-miR-888-5p	-0.013	1.000
TDGF1	hsa-miR-5696	-0.017	1.000
TDGF1	hsa-miR-466	0.146[a]	0.045[*]
TDGF1	hsa-miR-5585-3p	0	1.000
TDGF1	hsa-miR-4474-5p	0.075	0.813
TDGF1	hsa-miR-4538	-0.002	1.000
TDGF1	hsa-miR-4453	-0.064	0.822
TDGF1	hsa-miR-299-5p	-0.007	1.000
TDGF1	hsa-miR-3190-5p	-0.026	1.000
TDGF1	hsa-miR-6132	0.003	1.000
TDGF1	hsa-miR-202-3p	-0.028	1.000
TDGF1	hsa-miR-30e-3p	-0.041	1.000
TDGF1	hsa-miR-30d-3p	0.007	1.000
TDGF1	hsa-miR-766-5p	-0.088	0.602
TDGF1	hsa-miR-658	-0.017	1.000
TDGF1	hsa-miR-3614-5p	-0.205[a]	0.001[**]
TDGF1	hsa-miR-630	0.012	1.000
TDGF1	hsa-miR-4709-5p	-0.059	0.860
TDGF1	hsa-miR-4650-3p	0.003	1.000
TDGF1	hsa-miR-4457	-0.07	0.822
TDGF1	hsa-miR-4477a	0	1.000
TDGF1	hsa-miR-1244	0	1.000
TDGF1	hsa-miR-1283	-0.062	0.822
TDGF1	hsa-miR-4261	0	1.000
TDGF1	hsa-miR-3147	0	1.000
TDGF1	hsa-miR-4263	0	1.000
TDGF1	hsa-miR-1323	-0.023	1.000
TDGF1	hsa-miR-6128	0	1.000
TDGF1	hsa-miR-548o-3p	0.006	1.000
TDGF1	hsa-miR-5689	0	1.000
TDGF1	hsa-miR-3168	-0.052	0.909

TDGF1	hsa-miR-4693-5p	0	1.000
TDGF1	hsa-miR-5706	-0.055	0.909
TDGF1	hsa-miR-4782-5p	-0.036	1.000
TDGF1	hsa-miR-455-3p	-0.039	1.000

[a]These results are statistically significant.

[*]p value < 0.05; [**]p value < 0.01.

Table S3. Correlation analysis between hsa-miR-3614-5p and lncRNAs determined by ENCORI database

miRNA	lncRNAs	R value	Adjusted p value
hsa-miR-3614-5p	CBR3-AS1	-0.037	0.537
	EBLN3P	0.12[a]	0.037*
	FOXP1-IT1	0.076	0.212
	GABPB1-AS1	0.007[a]	0.908
	GABPB1-IT1	0.124[a]	0.037*
	GAS1RR	0.06	0.300
	HCG18	0.101	0.084
	HIPK1-AS1	0.062	0.293
	KCNQ1OT1	0.063	0.293
	KRTAP5-AS1	0.071	0.235
	LINC01089	-0.119[a]	0.037*
	LINC01128	0.121[a]	0.037*
	LINC01395	0.005	0.908
	LINC01714	0.054	0.349
	LINC02188	0.027	0.631
	LINC02288	0.124[a]	0.037*
	LOXL1-AS1	0.082	0.189
	MCM3AP-AS1	0.124[a]	0.037*
	MIR503HG	0.006	0.908
	MUC20-OT1	0.074	0.222
	NEAT1	0.168[a]	0.006**
	OLMALINC	-0.105[a]	0.047*
	PGR-AS1	0.027	0.631
	POLR2J4	-0.039	0.537
	PRR34-AS1	0.157[a]	0.009**
	PTPRG-AS1	0.037	0.537
	RGMB-AS1	0.093	0.120
	SNHG3	0.035	0.548
	STAG3L5P-PVRIG2P-PILRB	0.065	0.281
	TPT1-AS1	-0.079	0.201
	TUG1	0.18[a]	0.004**
	ZFHX2-AS1	0.125[a]	0.037*

[a]These results are statistically significant.

*p value < 0.05; **p value < 0.01.

Table S4. Correlation analysis between TDGF1 and lncRNAs determined by ENCORI database

Gene	lncRNAs	R value	Adjusted p value
TDGF1	CBR3-AS1	0.289[a]	0.000**
	EBLN3P	0.07	0.203
	FOXP1-IT1	-0.137[a]	0.007**
	GABPB1-AS1	0.06	0.264
	GABPB1-IT1	0.014	0.793
	GAS1RR	-0.142[a]	0.005**
	HCG18	0.197[a]	0.000**
	HIPK1-AS1	-0.049	0.367
	KCNQ1OT1	-0.062	0.263
	KRTAP5-AS1	0.253[a]	0.000**
	LINC01089	0.179[a]	0.000**
	LINC01128	-0.177[a]	0.000**
	LINC01395	-0.006	0.892
	LINC01714	-0.021	0.715
	LINC02188	-0.039	0.489
	LINC02288	-0.104[a]	0.047*
	LOXL1-AS1	-0.349[a]	0.000**
	MCM3AP-AS1	0.062	0.263
	MIR503HG	0.082	0.132
	MUC20-OT1	0.037	0.505
	NEAT1	-0.164[a]	0.001**
	OLMALINC	0.356[a]	0.000**
	PGR-AS1	-0.025	0.671
	POLR2J4	0.081	0.136
	PRR34-AS1	-0.017	0.769
	PTPRG-AS1	0.134[a]	0.007**
	RGMB-AS1	0.181[a]	0.000**
	SNHG3	-0.137[a]	0.007**
	STAG3L5P-PVRIG2P-PILRB	0.148[a]	0.004**
	TPT1-AS1	0.249[a]	0.000**
	TUG1	0.088	0.108
	ZFHX2-AS1	-0.049	0.367

[a]These results are statistically significant.

*p value < 0.05; **p value < 0.01.

Table S5. TDGF1 co-expression genes in colon cancer analyzed by LinkedOmics database

Query	Statistic (> 0.5)	p value (< 0.05)	FDR (BH) (<0 .05)	Event_SD	Event_TD
TDGF1	1	1E-32	1E-28	106	106
LRRC2	0.860790404	2.8257E-32	1.9048E-28	106	106
RNF43	0.697032513	1.05045E-16	4.7207E-13	106	106
AXIN2	0.687043701	4.23298E-16	1.42673E-12	106	106
LY6G6D	0.681702511	8.71904E-16	1.85923E-12	106	90
POU5F1B	0.681138428	9.40209E-16	1.85923E-12	106	106
AIFM3	0.680940914	9.65332E-16	1.85923E-12	106	106
PMFBP1	0.678904316	1.26544E-15	2.13259E-12	106	106
CDHR1	0.675140089	2.07529E-15	3.10879E-12	106	106
PLCB4	0.67353274	2.55774E-15	3.44835E-12	106	106
LOC101928881	0.666420817	6.35026E-15	7.78311E-12	106	106
PRLR	0.66381684	8.80463E-15	9.892E-12	106	106
NEBL	0.660768915	1.28542E-14	1.23786E-11	106	106
LFNG	0.659714383	1.46372E-14	1.25981E-11	106	106
IHH	0.659541764	1.49511E-14	1.25981E-11	106	106
ASCL2	0.655892466	2.3337E-14	1.85076E-11	106	106
VIL1	0.655359397	2.48926E-14	1.86445E-11	106	106
RNF114	0.654130489	2.88711E-14	2.04863E-11	106	106
GRM8	0.649625722	4.94298E-14	3.33206E-11	106	106
LY6G6E	0.648700332	5.51413E-14	3.54007E-11	106	85
CFTR	0.647312784	6.49197E-14	3.84633E-11	106	106
TUSC8	0.647221663	6.56175E-14	3.84633E-11	106	104
LINC01006	0.646781087	6.90952E-14	3.88142E-11	106	104
LINC01123	0.645367352	8.15023E-14	4.39526E-11	106	106
PLA2G4F	0.643581519	1.00285E-13	5.00756E-11	106	106
IYD	0.641494602	1.27567E-13	6.14234E-11	106	106
PTPRO	0.640451537	1.4377E-13	6.46104E-11	106	106
QPRT	0.639546333	1.59432E-13	6.93377E-11	106	106
LOC441242	0.634351331	2.86715E-13	1.20797E-10	106	106
CDX2	0.633309736	3.22089E-13	1.30204E-10	106	106
OXGR1	0.63299123	3.33724E-13	1.30204E-10	106	105
PPFIBP2	0.632876415	3.38017E-13	1.30204E-10	106	106
RUBCNL	0.631516209	3.93123E-13	1.47225E-10	106	106
GPR143	0.629590731	4.86214E-13	1.77166E-10	106	106
SHROOM4	0.623127496	9.81784E-13	3.48327E-10	106	106
CELP	0.622652614	1.03315E-12	3.57151E-10	106	96
ATP9A	0.620856046	1.25201E-12	4.11697E-10	106	106
AMACR	0.620619911	1.28391E-12	4.12136E-10	106	106
FOXP4-AS1	0.619336161	1.47159E-12	4.60096E-10	106	106
LOC100506127	0.619145844	1.50157E-12	4.60096E-10	106	106

ILDR1	0.616422928	2.001E-12	5.86468E-10	106	106
POFUT1	0.614534933	2.43786E-12	6.99303E-10	106	106
TNNC2	0.613893852	2.60616E-12	7.32004E-10	106	106
NR1I2	0.613412175	2.73993E-12	7.53873E-10	106	106
ACSL6	0.611496178	3.3408E-12	8.83151E-10	106	106
WDR77	0.611207738	3.44163E-12	8.92308E-10	106	106
THUMPD3-AS1	0.609300945	4.18602E-12	1.06483E-09	106	106
SPATA25	0.608867285	4.37585E-12	1.0925E-09	106	105
GTF2IRD2	0.607023199	5.28009E-12	1.29429E-09	106	106
DPEP1	0.605921197	5.90394E-12	1.37875E-09	106	106
PAAF1	0.605875259	5.93143E-12	1.37875E-09	106	106
VAV3	0.604889556	6.55205E-12	1.4972E-09	106	106
ANO9	0.603615265	7.4478E-12	1.67352E-09	106	106
ACOX2	0.599402006	1.13315E-11	2.50444E-09	106	106
DHRS12	0.599000594	1.179E-11	2.56375E-09	106	106
ZDHHC23	0.598681711	1.21669E-11	2.60372E-09	106	106
TRABD2A	0.597836021	1.32237E-11	2.78566E-09	106	106
CDCA7	0.597291366	1.39507E-11	2.89359E-09	106	106
GJB1	0.597099355	1.4216E-11	2.90395E-09	106	106
NAALADL2	0.59690846	1.44847E-11	2.91466E-09	106	106
REPS2	0.595321162	1.69162E-11	3.25807E-09	106	106
RPIA	0.593500318	2.01912E-11	3.67863E-09	106	106
LINC01106	0.59273763	2.17377E-11	3.86995E-09	106	106
NKD1	0.592161199	2.29817E-11	4.02389E-09	106	106
CCDC183	0.591972387	2.34039E-11	4.04528E-09	106	106
SNHG18	0.589704531	2.90972E-11	4.84306E-09	106	106
CADPS	0.587148929	3.71139E-11	5.91722E-09	106	106
VAPB	0.58633524	4.0086E-11	6.21195E-09	106	106
CTTNBP2	0.586121677	4.09033E-11	6.26658E-09	106	106
TFCP2L1	0.585674932	4.26653E-11	6.46307E-09	106	106
MOCS3	0.585433775	4.36466E-11	6.47653E-09	106	106
DNAH14	0.585417184	4.37149E-11	6.47653E-09	106	106
EFNA4	0.584574292	4.7324E-11	6.93503E-09	106	106
MAP1LC3A	0.58380586	5.08633E-11	7.37353E-09	106	106
FAAH	0.583599053	5.18585E-11	7.43784E-09	106	106
SEMA5A	0.583448771	5.25935E-11	7.46385E-09	106	106
EPHB2	0.581680492	6.20317E-11	8.62177E-09	106	106
GPSM2	0.580683575	6.80514E-11	9.26737E-09	106	106
B3GNT8	0.580274137	7.06835E-11	9.52955E-09	106	106
ADD3-AS1	0.578347921	8.44396E-11	1.12714E-08	106	106
METAP1D	0.576760118	9.76854E-11	1.28136E-08	106	106
CASC21	0.576736873	9.78935E-11	1.28136E-08	106	93
CAPN10-AS1	0.575420476	1.10398E-10	1.41755E-08	106	106

SLC35C2	0.575420129	1.10401E-10	1.41755E-08	106	106
AVL9	0.575231602	1.12313E-10	1.4285E-08	106	106
PXMP4	0.57465484	1.18363E-10	1.47757E-08	106	106
LOC100130705	0.574353737	1.21645E-10	1.50461E-08	106	106
DACH1	0.574213294	1.23206E-10	1.51006E-08	106	106
LOC100190940	0.574052022	1.25022E-10	1.51851E-08	106	96
ENGASE	0.573122822	1.35997E-10	1.63706E-08	106	106
FITM2	0.57294933	1.38146E-10	1.639E-08	106	106
JHDM1D-AS1	0.572655315	1.41863E-10	1.66313E-08	106	106
MOGAT3	0.571839169	1.52694E-10	1.77468E-08	106	106
CYP2B6	0.571682225	1.54866E-10	1.78454E-08	106	103
SYT7	0.56987323	1.82151E-10	2.06367E-08	106	106
BCL11A	0.568733321	2.01661E-10	2.24694E-08	106	106
ZSWIM3	0.568317668	2.09263E-10	2.29373E-08	106	106
SDR42E1	0.567020811	2.34797E-10	2.55285E-08	106	106
KIF12	0.565089396	2.78457E-10	2.97949E-08	106	106
LRP4	0.564943531	2.82054E-10	2.99421E-08	106	106
CXCL14	0.562844953	3.39023E-10	3.57087E-08	106	106
TMTC4	0.561982898	3.65501E-10	3.81991E-08	106	106
SLC19A3	0.561466823	3.82292E-10	3.95337E-08	106	106
DAPK2	0.560047525	4.32384E-10	4.3503E-08	106	106
YAE1D1	0.55960889	4.49102E-10	4.48503E-08	106	106
RXFP4	0.559027231	4.72232E-10	4.68135E-08	106	105
DNAJC3-AS1	0.558872405	4.78579E-10	4.70964E-08	106	106
ATP1A1	0.558178703	5.0804E-10	4.92762E-08	106	106
PLBD1-AS1	0.557611974	5.33394E-10	5.10016E-08	106	105
FANCF	0.557093343	5.5766E-10	5.29463E-08	106	106
SLC39A5	0.55603612	6.10453E-10	5.68761E-08	106	106
GOLIM4	0.556012092	6.11707E-10	5.68761E-08	106	106
ZNF41	0.555697962	6.28331E-10	5.80216E-08	106	106
NEK3	0.555353408	6.47063E-10	5.90832E-08	106	106
GATD1	0.555325727	6.48591E-10	5.90832E-08	106	106
BRCC3	0.553686698	7.4552E-10	6.7457E-08	106	106
TMEM147-AS1	0.553428804	7.61986E-10	6.80529E-08	106	106
ZNRF3	0.553425467	7.62201E-10	6.80529E-08	106	106
PIGZ	0.553149635	7.80204E-10	6.9202E-08	106	106
NEURL1B	0.552178301	8.4691E-10	7.40023E-08	106	106
LOC100996634	0.552124085	8.50791E-10	7.40023E-08	106	105
LY75	0.551341622	9.08733E-10	7.7654E-08	106	106
TIMM10B	0.551157232	9.22929E-10	7.79119E-08	106	106
VWA2	0.549120282	1.09463E-09	8.99864E-08	106	106
ACTL10	0.548469308	1.15569E-09	9.44305E-08	106	106
CXorf23	0.548383224	1.16401E-09	9.45371E-08	106	106

SLC7A6	0.548215378	1.18039E-09	9.47261E-08	106	106
HNF1A-AS1	0.547509407	1.25174E-09	9.98577E-08	106	106
SPIRE2	0.547265818	1.27731E-09	1.00706E-07	106	106
DDX27	0.544858503	1.55848E-09	1.20631E-07	106	106
RABEP2	0.544801377	1.56582E-09	1.20631E-07	106	106
STX3	0.544446273	1.61223E-09	1.23501E-07	106	106
IL17RE	0.544185637	1.64714E-09	1.24386E-07	106	106
CAPN13	0.544153691	1.65146E-09	1.24386E-07	106	105
OVGP1	0.543532559	1.73781E-09	1.30162E-07	106	106
A1CF	0.542568623	1.88044E-09	1.39297E-07	106	106
GID8	0.541809287	2.00065E-09	1.46591E-07	106	106
MANSC1	0.54163092	2.02993E-09	1.47933E-07	106	106
LOC100129046	0.541374028	2.07284E-09	1.50247E-07	106	106
USH1C	0.540496165	2.22612E-09	1.59641E-07	106	106
PEX16	0.539695702	2.37533E-09	1.6944E-07	106	106
ZDHHC9	0.539573323	2.39897E-09	1.70226E-07	106	106
QTRT1	0.539330427	2.44656E-09	1.72694E-07	106	106
OVOL1	0.537436236	2.85008E-09	1.99092E-07	106	106
ZNF513	0.536985802	2.95503E-09	2.04306E-07	106	106
MYRIP	0.536825559	2.99326E-09	2.04848E-07	106	106
PPP1R14D	0.536415585	3.09322E-09	2.09562E-07	106	106
ARFGEF2	0.535819692	3.24425E-09	2.18695E-07	106	106
SGK2	0.535531299	3.31986E-09	2.22679E-07	106	106
RBBP8NL	0.53333084	3.95507E-09	2.61384E-07	106	106
ME3	0.532696636	4.15879E-09	2.73506E-07	106	106
ANKDD1B	0.532418176	4.25139E-09	2.78239E-07	106	106
CEBPA-AS1	0.532297498	4.29212E-09	2.79548E-07	106	106
CEACAM5	0.532116928	4.35378E-09	2.822E-07	106	106
NELFCD	0.531524837	4.56197E-09	2.91441E-07	106	106
SCAP	0.530418624	4.97678E-09	3.12079E-07	106	106
RNF32	0.529616865	5.29977E-09	3.30794E-07	106	106
GTF2IRD2B	0.529284821	5.43934E-09	3.37941E-07	106	106
OSER1-AS1	0.528479355	5.79269E-09	3.55741E-07	106	106
SMAP1	0.52845214	5.80501E-09	3.55741E-07	106	106
PRR5L	0.528248009	5.89818E-09	3.59816E-07	106	106
NHLRC1	0.527990596	6.01772E-09	3.65455E-07	106	106
PRDX5	0.527679364	6.16536E-09	3.72741E-07	106	106
DUSP16	0.5271001	6.44943E-09	3.88175E-07	106	106
NSUN5P1	0.526784603	6.60938E-09	3.96034E-07	106	106
SELENBP1	0.525828797	7.11749E-09	4.24593E-07	106	106
CACNA1D	0.525618214	7.23437E-09	4.29664E-07	106	106
GPX2	0.52470576	7.76237E-09	4.56997E-07	106	106
LINC01558	0.524632261	7.80647E-09	4.57595E-07	106	106
TMPRSS13	0.524160517	8.0953E-09	4.72471E-07	106	106

GPR35	0.523811817	8.31534E-09	4.83222E-07	106	106
LINC01560	0.52373518	8.36446E-09	4.8399E-07	106	106
SECISBP2	0.521809062	9.69442E-09	5.52995E-07	106	106
PHOSPHO2	0.52175827	9.73209E-09	5.52995E-07	106	106
SLC5A6	0.521736217	9.7485E-09	5.52995E-07	106	106
CRCP	0.521717931	9.76212E-09	5.52995E-07	106	106
PTGES3L	0.521189278	1.01638E-08	5.70954E-07	106	106
SPATA2	0.520905664	1.03858E-08	5.81004E-07	106	106
ABAT	0.520771624	1.04924E-08	5.81305E-07	106	106
GPR160	0.520567054	1.0657E-08	5.86438E-07	106	106
RASSF10	0.520219147	1.09426E-08	5.99707E-07	106	103
LOC102723354	0.518695424	1.22822E-08	6.62203E-07	106	106
HNF4A	0.518645629	1.23285E-08	6.62203E-07	106	106
SSSCA1-AS1	0.518196266	1.27542E-08	6.79652E-07	106	106
EPB41L4B	0.518131051	1.28171E-08	6.80317E-07	106	106
ZBTB38	0.517250182	1.36969E-08	7.24161E-07	106	106
TCF7	0.517175225	1.37743E-08	7.25412E-07	106	106
SPIN3	0.516244436	1.4772E-08	7.74925E-07	106	106
ANKH	0.516152369	1.48743E-08	7.7727E-07	106	106
GATA6-AS1	0.515862681	1.52008E-08	7.91265E-07	106	106
GAS2	0.515125815	1.60625E-08	8.30293E-07	106	106
ANKS4B	0.515116475	1.60738E-08	8.30293E-07	106	106
KIF3B	0.514782989	1.64792E-08	8.44763E-07	106	106
SFXN5	0.514572001	1.67408E-08	8.51694E-07	106	106
STK38	0.514387563	1.69726E-08	8.58616E-07	106	106
GYG2	0.514362659	1.70042E-08	8.58616E-07	106	106
HUNK	0.514154505	1.72701E-08	8.67738E-07	106	106
ST6GAL1	0.514021281	1.74423E-08	8.67738E-07	106	106
NOX1	0.512802969	1.90956E-08	9.39586E-07	106	106
JADE3	0.511398957	2.11868E-08	1.03869E-06	106	106
KNOP1	0.510566593	2.25282E-08	1.09254E-06	106	106
LINC00654	0.510309643	2.29584E-08	1.10941E-06	106	106
PPP1R26-AS1	0.510063061	2.33787E-08	1.12503E-06	106	106
UPF3A	0.510022521	2.34485E-08	1.12503E-06	106	106
CACFD1	0.509691213	2.40264E-08	1.14058E-06	106	106
WDR5B	0.50888949	2.54817E-08	1.2042E-06	106	106
ACSL5	0.508588178	2.60501E-08	1.22372E-06	106	106
SEMA3C	0.508459403	2.62967E-08	1.22676E-06	106	106
WNK4	0.508066235	2.70636E-08	1.25529E-06	106	106
FREM1	0.508050579	2.70946E-08	1.25529E-06	106	106
FRMD1	0.50771508	2.77666E-08	1.27764E-06	106	106
CBFA2T2	0.507339405	2.85379E-08	1.30867E-06	106	106
CARF	0.507092933	2.90551E-08	1.32787E-06	106	106
SATB2-AS1	0.506915873	2.94321E-08	1.33604E-06	106	97

TP53RK	0.506399617	3.05583E-08	1.379E-06	106	106
SATB2	0.506388497	3.0583E-08	1.379E-06	106	106
SIRT5	0.505802823	3.19115E-08	1.4341E-06	106	106
CDH17	0.505528349	3.25529E-08	1.45504E-06	106	106
CCDC24	0.50547038	3.26899E-08	1.45504E-06	106	106
SLC25A13	0.504946975	3.39524E-08	1.49552E-06	106	106
PROX1	0.504730963	3.44869E-08	1.50959E-06	106	106
PHF20	0.504179976	3.5887E-08	1.56074E-06	106	106
ASB13	0.502976895	3.91352E-08	1.68569E-06	106	106
SHROOM2	0.502477876	4.05633E-08	1.74164E-06	106	105
ACOT11	0.501461475	4.36275E-08	1.85548E-06	106	106
CYP39A1	0.500156511	4.78869E-08	2.00567E-06	106	106
ENOX2	0.500151864	4.79028E-08	2.00567E-06	106	106

**Query**	**Statistic (< -0.5)**	**p value (< 0.05)**	**FDR (BH) (< 0.05)**	**Event_SD**	**Event_TD**
MAPK12	-0.66131754	1.20117E-14	1.23786E-11	106	106
DUSP4	-0.644898076	8.60785E-14	4.4635E-11	106	106
RELL1	-0.641128254	1.33045E-13	6.18523E-11	106	106
HPSE	-0.621490178	1.17008E-12	3.94375E-10	106	106
SMCHD1	-0.617273454	1.82989E-12	5.48235E-10	106	106
SOCS6	-0.613144173	2.81721E-12	7.59633E-10	106	106
FECH	-0.606018908	5.84587E-12	1.37875E-09	106	106
CD55	-0.596237069	1.54689E-11	3.06694E-09	106	106
CCDC68	-0.596064543	1.57321E-11	3.07391E-09	106	106
HIF1A	-0.594189705	1.88851E-11	3.56231E-09	106	106
NDEL1	-0.594114058	1.90244E-11	3.56231E-09	106	106
PITPNC1	-0.593850796	1.95167E-11	3.60444E-09	106	106
LPCAT1	-0.592700692	2.18155E-11	3.86995E-09	106	106
TBC1D8	-0.59156869	2.43318E-11	4.15243E-09	106	106
JAK2	-0.589846451	2.87048E-11	4.83748E-09	106	106
KCTD1	-0.588872569	3.15034E-11	5.17962E-09	106	106
ANTXR2	-0.588477987	3.27106E-11	5.31331E-09	106	106
ASPHD2	-0.587094363	3.73063E-11	5.91722E-09	106	106
FAM46A	-0.586432891	3.97176E-11	6.21195E-09	106	106
RAB27B	-0.583185546	5.39051E-11	7.5703E-09	106	106
RNF125	-0.580810432	6.72553E-11	9.2524E-09	106	106
PLK2	-0.574705096	1.17824E-10	1.47757E-08	106	106
INPP1	-0.572913902	1.38589E-10	1.639E-08	106	106
ZNF165	-0.570583872	1.70922E-10	1.95286E-08	106	106
UBE2G1	-0.568885152	1.98951E-10	2.23521E-08	106	106
RHOF	-0.568387348	2.0797E-10	2.29373E-08	106	106
KDSR	-0.566855369	2.38263E-10	2.56981E-08	106	106
UBASH3B	-0.561411486	3.84135E-10	3.95337E-08	106	106

CD274	-0.560803387	4.04969E-10	4.13621E-08	106	106
PPP4R1	-0.560429753	4.18305E-10	4.24029E-08	106	106
TYMP	-0.558723612	4.84757E-10	4.73586E-08	106	106
TOR1AIP2	-0.557954392	5.17933E-10	4.9877E-08	106	106
PLCL2	-0.556903552	5.66802E-10	5.3438E-08	106	106
FSCN1	-0.552438577	8.28517E-10	7.3007E-08	106	106
HS3ST1	-0.551949773	8.63383E-10	7.46162E-08	106	106
MAP3K5	-0.551324367	9.10052E-10	7.7654E-08	106	106
TRNP1	-0.551135287	9.24633E-10	7.79119E-08	106	106
STRIP2	-0.550700727	9.58998E-10	8.03057E-08	106	106
ANP32E	-0.549826748	1.03187E-09	8.58742E-08	106	106
TRIB2	-0.549428718	1.06679E-09	8.82358E-08	106	106
AHR	-0.548239766	1.17799E-09	9.47261E-08	106	106
MT2A	-0.547435688	1.25943E-09	9.98798E-08	106	106
DAPK1	-0.546129003	1.40341E-09	1.10004E-07	106	106
ADNP2	-0.544873225	1.55659E-09	1.20631E-07	106	106
APOL2	-0.544251102	1.6383E-09	1.24386E-07	106	106
STRN3	-0.543135625	1.79524E-09	1.3372E-07	106	106
CAPN2	-0.542117942	1.95092E-09	1.43728E-07	106	106
SERPINB8	-0.540819784	2.16839E-09	1.56333E-07	106	106
SLK	-0.538827033	2.5481E-09	1.78924E-07	106	106
SECTM1	-0.537121725	2.92298E-09	2.03132E-07	106	106
RARRES1	-0.536879694	2.98029E-09	2.04848E-07	106	106
CEP85L	-0.536613123	3.04466E-09	2.07314E-07	106	106
C19orf38	-0.534841172	3.50772E-09	2.34114E-07	106	106
VPS18	-0.533590713	3.87439E-09	2.57313E-07	106	106
MAPK11	-0.53202175	4.38662E-09	2.82969E-07	106	106
USP18	-0.531873373	4.43829E-09	2.84938E-07	106	106
IER5	-0.53144986	4.589E-09	2.91441E-07	106	106
MBP	-0.53138372	4.61298E-09	2.91441E-07	106	106
PIK3AP1	-0.531347806	4.62605E-09	2.91441E-07	106	106
TMEM263	-0.529129437	5.50586E-09	3.40504E-07	106	106
SPHK1	-0.525551646	7.27169E-09	4.29987E-07	106	106
ANXA1	-0.5231005	8.7821E-09	5.05984E-07	106	106
KCNK1	-0.52149913	9.9265E-09	5.59954E-07	106	106
SS18	-0.520745366	1.05134E-08	5.81305E-07	106	106
BARX2	-0.520736352	1.05206E-08	5.81305E-07	106	106
PNMA1	-0.519745353	1.13434E-08	6.17078E-07	106	106
ADGRG6	-0.519723468	1.13622E-08	6.17078E-07	106	106
CEP170	-0.519683347	1.13968E-08	6.17078E-07	106	106
EDEM1	-0.5183776	1.25807E-08	6.7307E-07	106	106
CRK	-0.515018248	1.61922E-08	8.33217E-07	106	106
RNASEK	-0.514604636	1.67E-08	8.51694E-07	106	106
ELL2	-0.514082924	1.73624E-08	8.67738E-07	106	106

PPM1K	-0.514063229	1.73879E-08	8.67738E-07	106	106
PMAIP1	-0.512874525	1.89945E-08	9.39266E-07	106	106
ARFGAP3	-0.512856839	1.90194E-08	9.39266E-07	106	106
WARS	-0.511282212	2.13702E-08	1.04389E-06	106	106
CCDC88A	-0.511215791	2.14753E-08	1.04523E-06	106	106
STK17B	-0.509871975	2.37094E-08	1.13351E-06	106	106
SLC39A6	-0.509771532	2.3885E-08	1.13787E-06	106	106
FUT8	-0.508855417	2.55454E-08	1.2042E-06	106	106
PRF1	-0.508468576	2.62791E-08	1.22676E-06	106	106
PKM	-0.507939585	2.73152E-08	1.26117E-06	106	106
TNFSF9	-0.506990892	2.92718E-08	1.33325E-06	106	106
RAB12	-0.505465636	3.27012E-08	1.45504E-06	106	106
TMX3	-0.505335487	3.30109E-08	1.46399E-06	106	106
ASPH	-0.505234417	3.32534E-08	1.46991E-06	106	106
PMP22	-0.504905443	3.40546E-08	1.49552E-06	106	106
LYN	-0.504275751	3.56398E-08	1.555E-06	106	106
ALKBH5	-0.503875078	3.66849E-08	1.59031E-06	106	106
SERPINB5	-0.50371888	3.71002E-08	1.60316E-06	106	106
MEX3C	-0.501694693	4.29055E-08	1.83341E-06	106	106
LMAN1	-0.501672791	4.29728E-08	1.83341E-06	106	106
GPAT3	-0.501279205	4.41999E-08	1.87136E-06	106	106
SAMD9L	-0.501254324	4.42786E-08	1.87136E-06	106	106
EZR	-0.50031427	4.73517E-08	1.99498E-06	106	106
COQ10A	-0.500091647	4.81087E-08	2.00805E-06	106	106

Table S6. TDGF1 co-expression genes in CRC single-cell samples analyzed by CancerSCEM database

Single-cell samples	Target Gene	Related Gene	Correlation Coefficient (Pearson)	Adjusted P-value
CRC-016-08-1A	TDGF1	CYP51A1	0.079	0
		KDM1A	0.051	0.022
		AOC1	0.045	0.038
		NDUFAB1	0.044	0.042
CRC-016-12-1A	TDGF1	GPRC5A	0.348	0
		SLC12A2	0.333	0
		TSPAN6	0.329	0
		SLC25A5	0.319	0
		TMEM176A	0.317	0
		ERBB3	0.313	0
		RPL18	0.308	0
		RPS20	0.298	0
		CD9	0.298	0
		PLEKHA5	0.29	0
		NFE2L3	0.289	0
		SH3YL1	0.283	0
		SPATA7	0.277	0
		MYLIP	0.272	0
		STRAP	0.272	0
		OGFR	0.259	0
		LETMD1	0.253	0
		LIMCH1	0.252	0
		DPM1	0.249	0
		SYPL1	0.249	0
		GUCY2C	0.247	0
		VMP1	0.245	0
		MYOM2	0.245	0
		VPS41	0.245	0
		TTC19	0.244	0
		GYG2	0.239	0
		CROT	0.235	0
		MRPS24	0.231	0
		MLXIPL	0.229	0
		PABPC1	0.228	0
		KCNAB2	0.225	0
		KRIT1	0.223	0
		RPAP3	0.222	0
		M6PR	0.22	0
		RNASET2	0.22	0

CRC-016-12-1A	TDGF1	TM7SF3	0.218	0
		VPS35	0.217	0
		GSTO2	0.217	0
		PRSS3	0.216	0
		PRSS22	0.215	0
		NTHL1	0.213	0
		OFD1	0.213	0
		RRP12	0.213	0
		TNPO3	0.212	0
		IL32	0.21	0
		CS	0.209	0
		EXTL3	0.209	0
		FKBP4	0.207	0
		SLC7A2	0.207	0
		MTMR1	0.207	0
		PHPT1	0.206	0
		EIF4B	0.205	0
		KDM7A	0.202	0
		CDH1	0.201	0
		DGKG	0.2	0
		TFE3	0.198	0
		CASR	0.195	0
		FARP2	0.194	0
		RTF2	0.194	0
		IDH3G	0.193	0.002
		ATP9A	0.192	0.002
		ATXN3	0.192	0.002
		SCML1	0.188	0.002
		RCN1	0.188	0.002
		HCCS	0.188	0.002
		RRAGD	0.186	0.002
		ABCC2	0.183	0.002
		ARSD	0.181	0.002
		CFTR	0.18	0.002
		RB1CC1	0.178	0.002
		PITX1	0.178	0.002
		FNIP2	0.176	0.004
		PLEKHG6	0.175	0.004
		OAT	0.175	0.004
		LAS1L	0.175	0.004
		PRSS8	0.174	0.004
		SERPINB1	0.174	0.004
		PIGB	0.172	0.004
		KMT2C	0.172	0.004

CRC-016-12-1A	TDGF1	PLEKHB1	0.171	0.004
		KMT2E	0.171	0.004
		RRP15	0.17	0.004
		TNFRSF12A	0.169	0.004
		LY75	0.169	0.004
		ZFYVE16	0.168	0.004
		CD44	0.167	0.006
		GABARAPL2	0.166	0.006
		CTSA	0.166	0.006
		LAMP2	0.166	0.006
		PTPN3	0.164	0.006
		CHDH	0.163	0.006
		VEZT	0.163	0.006
		EIF2AK2	0.163	0.006
		THOC3	0.162	0.007
		KITLG	0.162	0.006
		PROM1	0.162	0.007
		IDS	0.161	0.007
		POLR2J	0.16	0.007
		LPAR2	0.159	0.007
		UBE3C	0.159	0.007
		DIP2B	0.158	0.007
		CYFIP2	0.157	0.009
		YBX3	0.156	0.009
		BRD9	0.154	0.01
		BCAS1	0.154	0.01
		HEBP2	0.154	0.01
		SLC25A39	0.153	0.01
		ELAVL1	0.153	0.01
		ICA1	0.152	0.01
		COL17A1	0.15	0.012
		YBX1	0.147	0.013
		PTPN21	0.146	0.015
		PNPLA4	0.145	0.015
		MRPS35	0.144	0.016
		ZZZ3	0.143	0.016
		FAM120A	0.142	0.017
		PDK3	0.142	0.017
		TRAF3IP2	0.141	0.017
		HMGB3	0.14	0.018
		KCNG1	0.139	0.019
		GGCT	0.139	0.018
		ADAT1	0.139	0.019
		PIK3C2A	0.138	0.019

CRC-016-12-1A	TDGF1	HS3ST1	0.138	0.019
		NDUFB4	0.138	0.019
		CAPN1	0.137	0.02
		IFRD1	0.135	0.022
		FAR2	0.135	0.022
		ALS2	0.134	0.022
		MPHOSPH9	0.134	0.022
		BID	0.133	0.023
		OSBPL3	0.132	0.025
		HEATR5B	0.131	0.026
		SLC4A7	0.13	0.026
		CUL7	0.13	0.026
		NDUFAB1	0.13	0.026
		MRI1	0.129	0.027
		SLC25A13	0.129	0.026
		TMEM159	0.129	0.026
		PER3	0.128	0.027
		DNAJA2	0.127	0.029
		GRAMD1B	0.126	0.03
		RHOA	0.126	0.03
		SPHK2	0.125	0.03
		GRIPAP1	0.123	0.034
		NIPAL3	0.123	0.034
		CC2D2A	0.122	0.034
		PKD1	0.122	0.034
		ELAC2	0.121	0.035
		DSG2	0.121	0.037
		FOXJ2	0.121	0.036
		ZRANB1	0.12	0.038
		SS18L2	0.12	0.037
		PHF20	0.119	0.038
		ABCA7	0.119	0.038
		RC3H2	0.119	0.038
		POLB	0.117	0.041
		CDKL5	0.117	0.041
		ZFR	0.117	0.041
		IFT88	0.116	0.041
		WNK1	0.116	0.041
		XK	0.115	0.043
		AK2	0.115	0.043
		MNAT1	0.115	0.043
		DAPP1	0.115	0.043
		BAZ1B	0.115	0.043
		TMEM161A	0.114	0.045

CRC-016-12-1A	TDGF1	LIMA1	0.114	0.044
		RWDD2A	0.113	0.046
		ALDH18A1	0.113	0.045
		SPATA20	0.113	0.045
		DPEP1	0.112	0.046
		PDK2	0.111	0.047
		PIK3CB	0.111	0.047
		KCNH2	0.111	0.047
		PKN2	0.11	0.049
		WWC3	0.109	0.05
		INTS13	0.109	0.05
		NUCB2	-0.117	0.041
		RNF19A	-0.123	0.034
		POU2F2	-0.128	0.027
		ISOC2	-0.133	0.023
		CD79B	-0.144	0.016
		TRAM1	-0.151	0.012
		TYMP	-0.153	0.01
		SLAMF7	-0.156	0.009
		AGA	-0.162	0.007
		BIRC3	-0.165	0.006
		CD38	-0.175	0.004
		CYBA	-0.214	0
		PRDM1	-0.225	0
		VIM	-0.229	0
		TNFRSF17	-0.241	0
		HERPUD1	-0.247	0
		CD74	-0.25	0
CRC-016-13-1A	TDGF1	POLDIP2	0.177	0
		FKBP4	0.15	0
		NDUFAB1	0.148	0
		ICA1	0.146	0
		TSPAN6	0.116	0
		AOC1	0.09	0.009
		CYP51A1	0.083	0.016
		DPM1	0.075	0.026
		KLHL13	0.072	0.03
		CFTR	0.064	0.046
		PLXND1	-0.063	0.049
		M6PR	-0.064	0.046
		CFH	-0.065	0.045
		TFPI	-0.066	0.043
		LASP1	-0.068	0.038
		RBM6	-0.079	0.019

		CD38	-0.081	0.018
CRC-016-13-1A	TDGF1	CFLAR	-0.161	0
		CD99	-0.177	0

Table S7. Top 20 clusters in pathway and process enrichment analysis of TDGF1 associated DEGs in CRC single-cell samples analyzed by Metascape database

Cluster	Description	Log10(P)	Log10(q)
GO:0120035	regulation of plasma membrane bounded cell projection organization	-7.17	-2.94
GO:2001234	negative regulation of apoptotic signaling pathway	-6.27	-2.53
R-HSA-9012999	RHO GTPase cycle	-5.52	-2.02
GO:0033365	protein localization to organelle	-5.45	-2.01
R-HSA-9613829	Chaperone Mediated Autophagy	-4.85	-1.58
GO:0031330	negative regulation of cellular catabolic process	-4.82	-1.58
WP4754	IL-18 signaling pathway	-4.6	-1.43
R-HSA-6798695	Neutrophil degranulation	-4.52	-1.41
GO:0007005	mitochondrion organization	-4.46	-1.41
GO:0090066	regulation of anatomical structure size	-4.46	-1.41
hsa04140	Autophagy - animal	-4.25	-1.26
GO:0051046	regulation of secretion	-4.08	-1.21
GO:0003084	positive regulation of systemic arterial blood pressure	-4.06	-1.21
GO:0042176	regulation of protein catabolic process	-4.01	-1.2
GO:0097190	apoptotic signaling pathway	-3.98	-1.19
GO:0010959	regulation of metal ion transport	-3.94	-1.16
GO:0010876	lipid localization	-3.79	-1.11
GO:0008610	lipid biosynthetic process	-3.77	-1.11
GO:0048535	lymph node development	-3.62	-1.03
GO:0030155	regulation of cell adhesion	-3.59	-1.03

Note: "Log10(P)" is the p-value in log base 10. "Log10(q)" is the multi-test adjusted p-value in log base 10.
